# The Prognostic Accuracy of National Early Warning Score 2 on Predicting Clinical Deterioration for Patients With COVID-19: A Systematic Review and Meta-Analysis

**DOI:** 10.3389/fmed.2021.699880

**Published:** 2021-07-09

**Authors:** Kai Zhang, Xing Zhang, Wenyun Ding, Nanxia Xuan, Baoping Tian, Tiancha Huang, Zhaocai Zhang, Wei Cui, Huaqiong Huang, Gensheng Zhang

**Affiliations:** ^1^Department of Critical Care Medicine, Second Affiliated Hospital, Zhejiang University School of Medicine, Hangzhou, China; ^2^Medical Security Bureau of Yinzhou District, Ningbo, China; ^3^Department of Respiration and Critical Care Medicine, Second Affiliated Hospital, Zhejiang University School of Medicine, Hangzhou, China

**Keywords:** NEWS2, COVID-19, systematic review, meta-analysis, prediction

## Abstract

**Background:** During the coronavirus disease 2019 (COVID-19) pandemic, the National Early Warning Score 2 (NEWS2) is recommended for the risk stratification of COVID-19 patients, but little is known about its ability to detect severe cases. Therefore, our purpose is to assess the prognostic accuracy of NEWS2 on predicting clinical deterioration for patients with COVID-19.

**Methods:** We searched PubMed, Embase, Scopus, and the Cochrane Library from December 2019 to March 2021. Clinical deterioration was defined as the need for intensive respiratory support, admission to the intensive care unit, or in-hospital death. Sensitivity, specificity, and likelihood ratios were pooled by using the bivariate random-effects model. Overall prognostic performance was summarized by using the area under the curve (AUC). We performed subgroup analyses to assess the prognostic accuracy of NEWS2 in different conditions.

**Results:** Eighteen studies with 6,922 participants were included. The NEWS2 of five or more was commonly used for predicting clinical deterioration. The pooled sensitivity, specificity, and AUC were 0.82, 0.67, and 0.82, respectively. Benefitting from adding a new SpO_2_ scoring scale for patients with hypercapnic respiratory failure, the NEWS2 showed better sensitivity (0.82 vs. 0.75) and discrimination (0.82 vs. 0.76) than the original NEWS. In addition, the NEWS2 was a sensitive method (sensitivity: 0.88) for predicting short-term deterioration within 72 h.

**Conclusions:** The NEWS2 had moderate sensitivity and specificity in predicting the deterioration of patients with COVID-19. Our results support the use of NEWS2 monitoring as a sensitive method to initially assess COVID-19 patients at hospital admission, although it has a relatively high false-trigger rate. Our findings indicated that the development of enhanced or modified NEWS may be necessary.

## Introduction

The recent outbreak of coronavirus disease 2019 (COVID-19), caused by severe acute respiratory syndrome coronavirus 2 (SARS-CoV-2), has challenged healthcare systems worldwide (1). As of March 26, 2021, SARS-CoV-2 has results in more than 12.5 million confirmed cases, with more than 2.7 million deaths (2). Although the majority of patients infected with COVID-19 are symptomless or oligosymptomatic, about one-fifth of patients may develop severe COVID-19 with a high risk of mortality (3, 4). Thus, for patients with COVID-19, early identification of the deteriorating patients is of importance because it could direct finite resources toward those patients in greatest clinical need. However, risk stratification and early identification of patients with high risk of clinical deterioration at admission remain as major challenges. Frontline health workers constantly meet the challenges of determining the severity and prognosis of COVID-19 cases in order to provide high-quality care and effectively allocate resources (5). Therefore, there is a need for an easy-to-use and effective risk-predictive tool to assess the possibility of deterioration of patients with COVID-19.

The National Early Warning Score (NEWS), first introduced in 2012 and updated in 2017 (NEWS2), has received a formal endorsement from the National Health Service to become the early warning system for deterioration of acutely ill patients in the United Kingdom (UK) (6, 7). The NEWS/NEWS2 is a scoring system based on routine physiological parameters, which can be obtained easily and rapidly at the bedside. Each indicator is given a score, where 0 is considered normal, and simple addition allows a total score from 0 to 23. A score of 5 or more represents the key threshold for urgent response, and patients with a score of 7 or more would be deemed to have a high clinical risk and trigger a high-level clinical alert (Table 1) (6, 7). Since some components (e.g., temperature, oxygen saturation, and supplemental oxygen dependency) were proved to be associated with the progression of COVID-19 (8, 9), guidelines from the Royal College of Physicians (10) and the Swiss Society of Intensive Care Medicine (11) advocate the use of the NEWS2 for initial assessment in patients with COVID-19. However, these recommendations were only based on expert opinions, and there have been no published meta-analyses to evaluate the predictive performance of the NEWS2.

**Table 1:** The NEWS scoring system, thresholds, and triggers.

	**3**	**2**	**1**	**0**	**1**	**2**	**3**
**NEWS**								
Respiration rate	≤ 8		9–11	12–20		21–24	≥25
Oxygen saturations	≤ 91	92–93	94–95	≥96			
Any supplemental oxygen		Yes		No			
Temperature	≤ 35.0		35.1–36.0	36.1–38.0	38.1–39.0	≥39.1	
% Systolic blood pressure	≤ 90	91–100	101–110	111–219			≥220
Heart rate	≤ 40		41–50	51–90	91–110	111–130	≥131
Level of Consciousness				Alert			V, P, or U
**NEWS2**								
Respiration rate	≤ 8		9–11	12–20		21–24	≥25
SpO_2_ scale 1	≤ 91	92–93	94–95	≥96			
SpO_2_ scale 2	≤ 83	84–85	86–87	88–92 ≥93 on air	93–94 on oxygen	95–96 on oxygen	≥97 on oxygen
Air or oxygen?		Oxygen		Air			
Systolic blood pressure	≤ 90	91–100	101–110	111–219			
Pulse	≤ 40		41–50	51–90	91–110	111–130	≥131
Consciousness				Alert			CVPU
Temperature	≤ 35.0		35.1–36.0	36.1–38.0	38.1–39.0	≥39.1	
**Score**	**Clinical risk**			**Response**			
Aggregate score 0–4	Low			Ward-based response			
Score of 3 in any individual parameter	Low–medium			Urgent ward-based response			
Aggregate score 5–6	Medium			Key threshold for urgent response			
Aggregate score 7 or more	High			Urgent or emergency response			

Therefore, the aim of the present study was to evaluate the prognostic accuracy of the NEWS2 on predicting clinical deterioration for patients with COVID-19. In addition, we performed a comparison of the NEWS2 with the original NEWS.

## Methods

### Study Selection

We followed the PRISMA statement (12) to structure the meta-analysis (Supplementary Material 1). A predefined protocol has been registered in PROSPERO (CRD42021243845, Supplementary Material 2). We searched the PubMed, Embase, Scopus, and the Cochrane Library from December 2019 to March 2021 for relevant articles.

The basic inclusive criteria are as follows: (1) recruited adult patients with confirmed cases of SARS-CoV-2 infection, (2) applied the NEWS2 or the NEWS to predict clinical deterioration (including the need for intensive respiratory support, admission to the ICU, or in-hospital death), and (3) provided sufficient data to estimate the prognostic accuracy. There was no language restriction. The detailed searching strategies and inclusion and exclusion criteria are recorded in Supplementary Material 3.

### Data Extraction

Two authors independently retrieved and extracted studies according to the inclusion criteria. We recorded the true positive, false positive, false negative, and true negative from the articles directly or through a recalculation according to the sensitivity and specificity. Any disagreement in the process was resolved by a discussion.

### Quality Assessment

Two authors employed the PROBAST to assess the risk of bias and applicability concerns of the included studies (13). The detailed quality assessment standard is recorded in Supplementary Material 3.

### Statistical Synthesis and Analysis

We used a bivariate random-effects regression model (14) to pool the sensitivity, specificity, positive likelihood ratio (PLR), negative likelihood ratio (NLR), and area under the curve (AUC) as point estimates with 95% confidence interval (CI). We also constructed the hierarchical summary receiver operating characteristic (HSROC) curve to present the summary point estimates of sensitivity and specificity. *I*^2^ statistics were calculated to assess the statistical heterogeneity between the included studies, where *I*^2^ > 50% indicated a substantial level of heterogeneity (15).

We performed subgroup analyses to evaluate the performance of the NEWS2 in different conditions. Studies were stratified according to the time of outcome measurement (within 72 h vs. in-hospital) and disease severity (mortality rate <10 vs. ≥10%). Sensitivity analyses were conducted by repeating the analyses within studies that calculated the NEWS2 at hospital admission. Publication bias was evaluated by using the Deek's test for funnel plot asymmetry (16), with *p*-value < 0.1 indicating publication bias. All analyses were performed using Stata 14.0 (StataCorp LP, College Station, TX, USA) and Review Manager 5.3 (The Cochrane Collaboration).

## Results

### Study Selection and Characteristics

**Figure 1:**
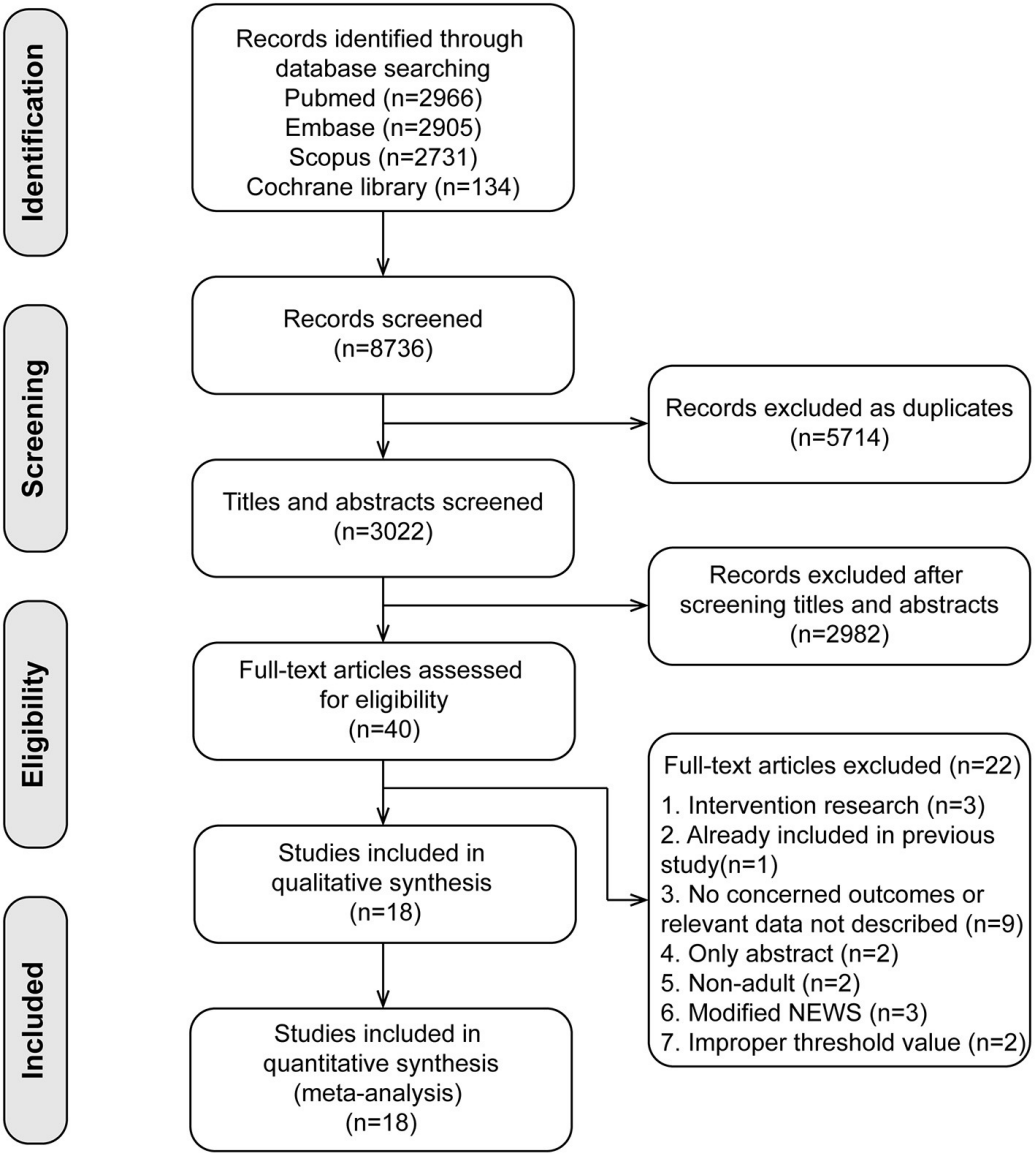
Flow diagram of study inclusion.

A total of 8,746 published studies were initially identified. After removing the duplicate articles and screening the abstracts, we identified 40 studies, and 22 studies were excluded with reasons in the full-text assessments (the list of excluded studies with reasons is shown in Supplementary Material 4). Finally, we included 18 studies (17–34) in our meta-analyses (Figure 1).

Table 2 shows the basic information and characteristics of the included studies. A total of 6,922 participants were included in the analysis, with the mortality rate in each study ranging from 6 to 47%. Three studies (22, 24, 28) were relatively small in sample size (<100), and six studies (17, 19, 21, 26, 30, 34) enrolled more than 400 patients. Fifteen studies (17–19, 21, 22, 24–27, 29–34) investigated general ward patients, and three (20, 23, 28) investigated only the emergency department (ED) population. Fourteen studies (18–26, 28, 30, 32–34) used the NEWS2, while another four (17, 27, 29, 31) studies only used the original NEWS. Moreover, in six studies (18, 19, 23, 24, 28, 31), the investigators employed a positive quick Sequential Organ Failure Assessment qSOFA (≥2) to predict clinical deterioration.

**Table 2:** Characteristics of the included studies.

**References**	**Sample size**	**Design**	**Participants**	**Score**	**Outcome**	**Time of calculating scores**
Aliberti et al. (17)	1,428	Prospective	Age: 66 (59, 74); male: 58%; 30-day mortality: 37%	NEWS ≥6	30-day mortality, 60-day mortality	At admission
Baker et al. (18)	296	Retrospective	Age: 75 (62, 84); male: 55%; in-hospital mortality: 26%	NEWS2 ≥5 qSOFA ≥2	Serious events with 24 h	Daily from admission until the occurrence of outcome
Bradley et al. (19)	830	Retrospective	Age: 70 (58, 80); male: 61%; 30-day mortality: 36%	NEWS2 ≥5 qSOFA ≥2	72-h mortality, 30-day mortality	Earliest measurement recorded after admission
Covino et al. (20)	334	Retrospective	Age: 66 (54, 78); male: 64%; 7-day mortality: 8%	NEWS ≥5 NEWS2 ≥5	ICU admission within 48 h, and 7 days	At ED arrival
De Socio et al. (32)	121	Retrospective	Age: 65 ± 13; male: 65%; mortality: NR	NEWS2 ≥4	ICU admission, invasive ventilation, or death	At admission
Fan et al. (21)	654	Retrospective	Age: NR; male: NR; in-hospital mortality: 20%	NEWS2 ≥5	In-hospital mortality	At admission
Gidari et al. (22)	68	Retrospective	Age: 64 (31, 93); male: 66%; mortality: NR	NEWS2 ≥5	ICU admission	At admission
Holten et al. (23)	169	Prospective	Age: 59; male: 58%; 14-day mortality: 7%	NEWS2 ≥5 qSOFA ≥2	Death or admission to ICU within 14 days	At ED arrival
Ihle-Hansen et al. (24)	42	Retrospective	Age: 73; male: 67%; in-hospital mortality: 47%	NEWS2 ≥5, qSOFA ≥2	Death or admission to ICU	First examination after admission
Jang et al. (25)	110	Retrospective	Age: 57 ± 17; male: 44%; 28-day mortality: 6%	NEWS2 ≥5	Death or admission to ICU	NR
Liu et al. (26)	673	Retrospective	Age: 61 (50, 69); male: 51%; in-hospital mortality: 18%	NEWS ≥5 NEWS2 ≥5	In-hospital mortality	At admission
Maguire et al. (27)	224	Retrospective	Age: NR; male: 55%; 30-day mortality: 23%	NEWS ≥5	30-day mortality	At admission
Martin-Rodriguez et al. (33)	261	Retrospective	Age: 80 (69, 88); male: 46%; 2-day mortality: 12%	NEWS2 ≥8	Death within 2 days	At admission
Myrstad et al. (28)	66	Prospective	Age: 72; male: 58%; in-hospital mortality: 20%	NEWS2 ≥5 qSOFA ≥2	Death or admission to ICU	At ED admission
Pokeerbux et al. (29)	202	Retrospective	Age: 65 (52, 78); male: 61%; in-hospital mortality: 11%	NEWS ≥5	Death or admission to ICU	At admission
Prower et al. (34)	708	Retrospective	Age: 62 ± 18; male: 58%; in-hospital mortality: 12%	NEWS2 ≥5	Death or admission to ICU	At admission
Richardson et al. (30)	620	Retrospective	Age: 73; male: 55%; in-hospital mortality: 32%	NEWS ≥5 NEWS2 ≥5	24-h mortality, in-hospital mortality	With 24 h of admission
Su et al. (31)	116	Retrospective	Age: 63 (51, 72); male: 48%; in-hospital mortality: 8%	NEWS ≥6 qSOFA ≥2	Need intensive respiratory support	At admission

*NEWS, National Early Warning Score; NEWS2, National Early Warning Score 2; qSOFA, quick Sequential Organ Failure Assessment; ICU, intensive care unit; ED, emergency department*.

### Quality Assessment

Table 3 shows the summary results of the quality assessments by using PROBAST. Overall, 16 studies had a high or unclear risk of bias, mainly because of the inappropriate handling method of missing data (11 studies excluded participants with missing values from the analyses, and five studies did not explicitly state the handling method of the missing data). Four studies had a high or unclear concern regarding applicability since the threshold value of the NEWS or the time interval between the evaluation of predictor and the determination of the outcome were not consistent with other studies. The details of the quality assessment are reported in Supplementary Material 5.

**Table 3:** PROBAST results.

**Study**	**ROB**				**Applicability**			**Overall**	
	**Participants**	**Predictors**	**Outcome**	**Analysis**	**Participants**	**Predictors**	**Outcome**	**ROB**	**Applicability**
Aliberti	–	+	+	?	+	–	+	–	–
Baker	+	+	–	–	+	+	+	–	+
Bradley	+	+	?	–	+	+	?	–	?
Covino	+	+	+	–	+	+	+	–	+
De Socio	+	+	+	–	+	+	+	–	+
Fan	?	+	+	–	+	+	+	–	+
Gidari	+	+	+	–	+	+	+	–	+
Holten	+	+	+	?	+	+	+	?	+
Ihle-Hansen	+	+	?	–	+	+	+	–	+
Jang	+	+	?	?	+	+	?	?	?
Liu	+	+	+	+	+	+	+	+	+
Maguire	+	+	+	–	+	+	+	–	+
Martín-Rodríguez	+	+	+	+	+	+	+	+	+
Mystad	+	+	+	–	+	+	+	–	+
Pokeerbux	+	+	+	?	+	+	+	?	+
Prower	+	+	+	?	+	+	+	?	+
Richardson	+	+	+	–	+	+	+	–	+
Su	+	+	+	?	+	–	+	–	–

*PROBAST, Prediction model Risk Of Bias ASsessment Tool; ROB, risk of bias*.

*“+” indicates low ROB/low concern regarding applicability; “–” indicates high ROB/high concern regarding applicability; and “?” indicates unclear ROB/unclear concern regarding applicability*.

In addition, the Deek's funnel plot indicated that there was a potential publication bias among the included studies (Deek's test: *P* < 0.10, Supplementary Material 6).

### Results of the Synthesis

**Figure 2:**
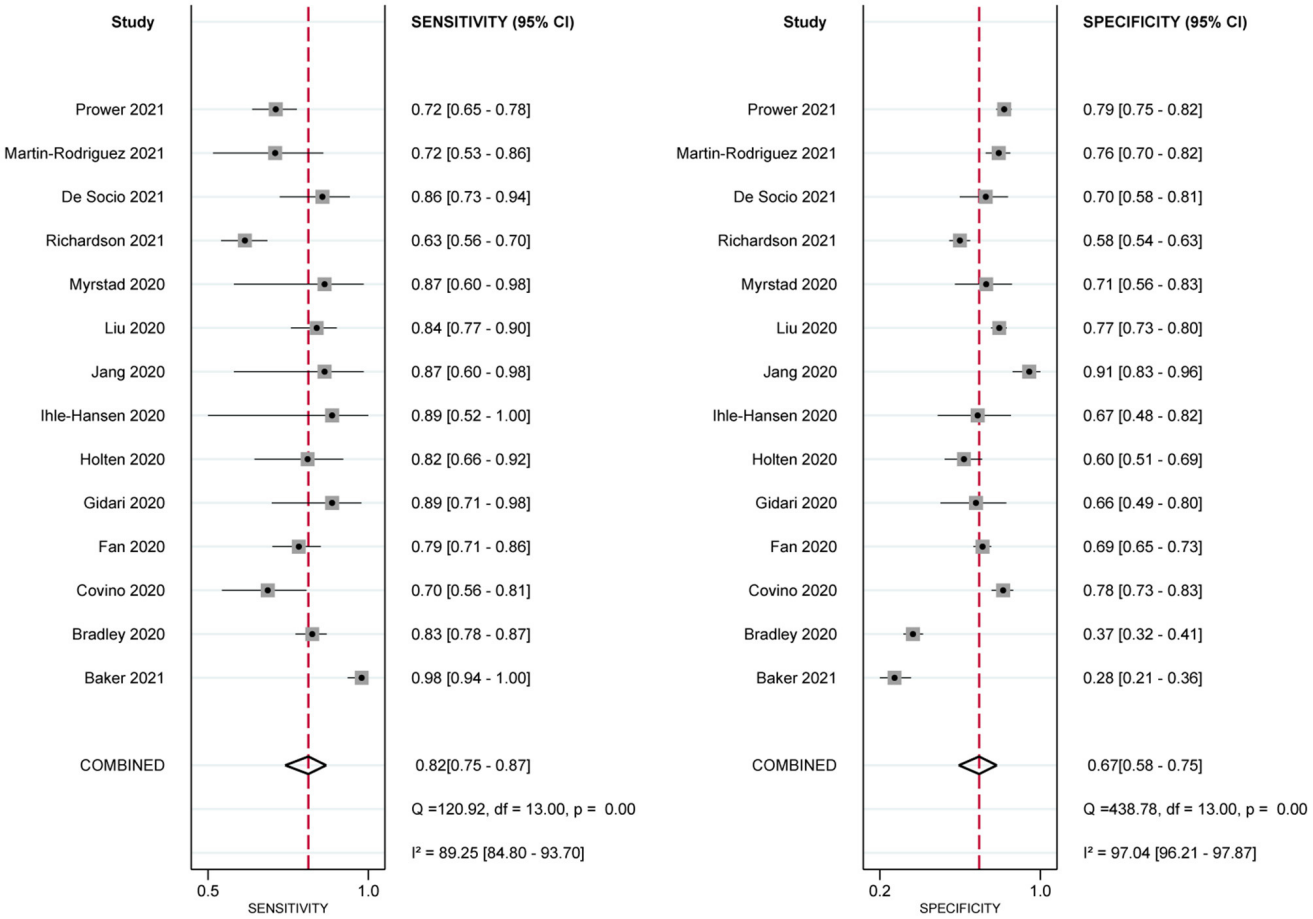
Paired forest plots of sensitivity and specificity of National Early Warning Score 2 (NEWS2) in predicting clinical deterioration in patients with COVID-19.

**Figure 3:**
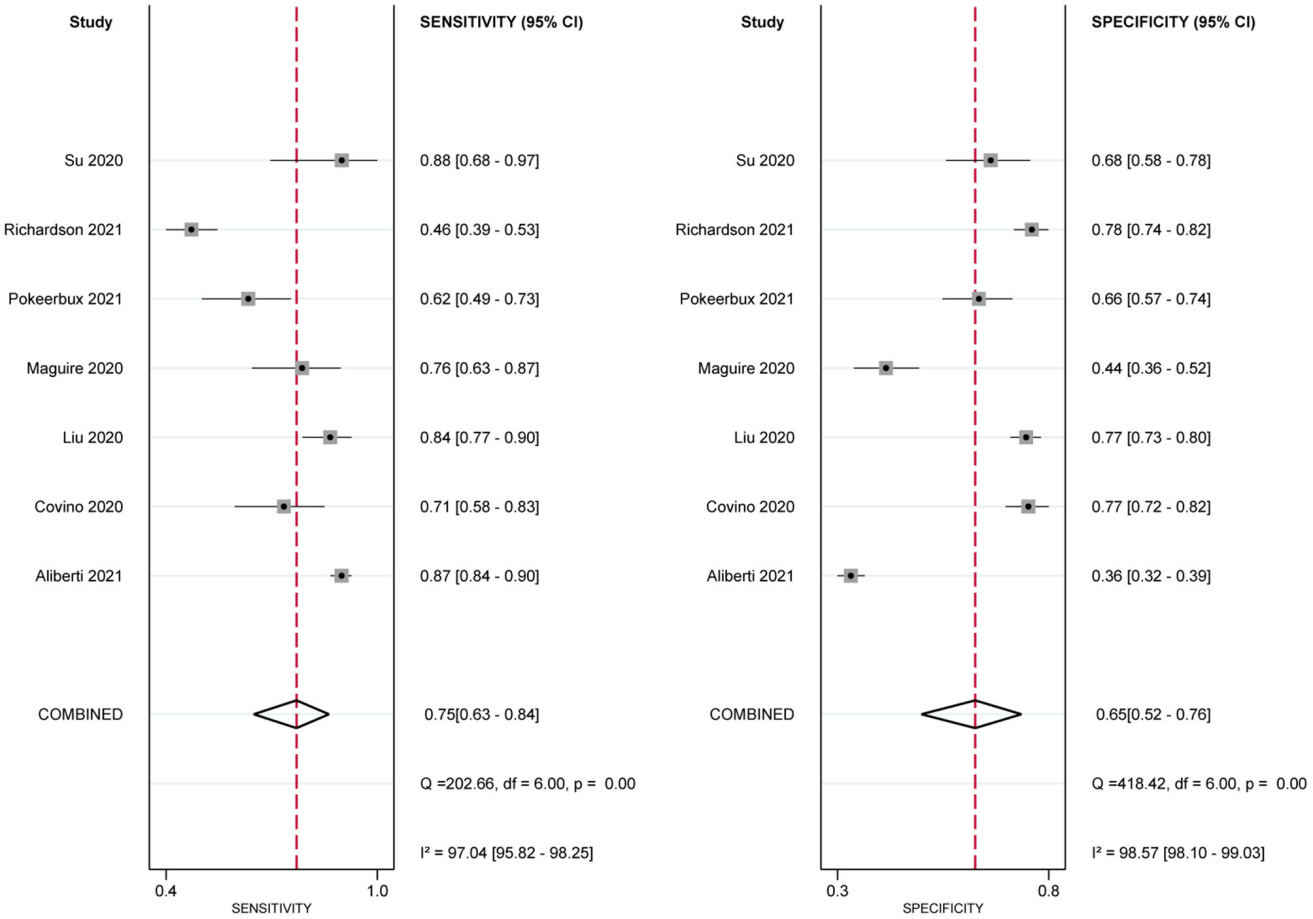
Paired forest plots of sensitivity and specificity of National Early Warning Score (NEWS) in predicting clinical deterioration in patients with COVID-19.

**Figure 4:**
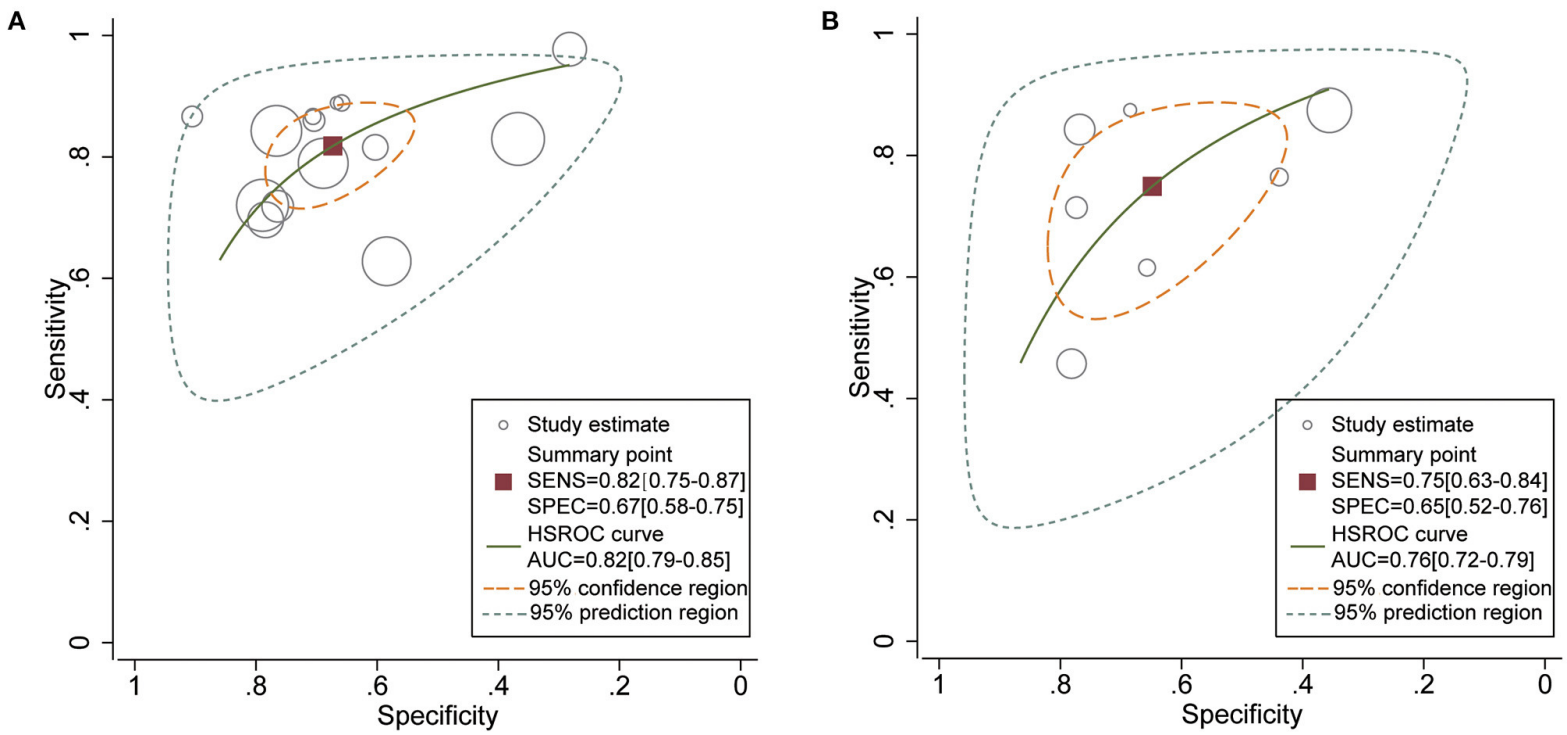
Hierarchical summary ROC curves for **(A)** National Early Warning Score 2 (NEWS2) and **(B)** National Early Warning Score (NEWS) for predicting clinical deterioration in patients with COVID-19.

Eleven studies used the NEWS2 to predict clinical deterioration for patients with COVID-19. Figure 2 shows the forest plot of sensitivity and specificity for the NEWS2; the pooled sensitivity and specificity of the NEWS2 were 0.82 (95% CI: 0.75, 0.87) and 0.67 (95% CI: 0.58, 0.75). The pooled PLR and NLR of the NEWS2 were 2.50 (95% CI: 1.96, 3.20) and 0.27 (95% CI: 0.20, 0.37). In seven studies reporting the prognostic accuracy of the NEWS (Figure 3), the pooled sensitivity and specificity were 0.75 (95% CI: 0.63, 0.84) and 0.65 (95% CI: 0.52, 0.76). Figure 4 shows the HSROC curves for the NEWS2 (Figure 4A) and the NEWS (Figure 4B); the AUC was 0.82 (95% CI: 0.79, 0.85) and 0.76 (95% CI: 0.72, 0.79), respectively. Considerable heterogeneity existed across the studies.

In six studies, the researchers employed the qSOFA to predict clinical deterioration. The pooled sensitivity, specificity, and AUC of qSOFA were 0.26 (95% CI: 0.20, 0.33), 0.94 (95% CI: 0.86, 0.97), and 0.64 (95% CI: 0.60, 0.80), respectively (Table 4).

**Table 4:** Results of the meta-analysis.

**Results**	***N***	**Sensitivity (95% CI)**	**Specificity (95% CI)**	**PLR (95% CI)**	**NLR (95% CI)**	**AUC (95% CI)**
NEWS2	14	0.82 (0.75, 0.87)	0.67 (0.58, 0.75)	2.50 (1.96, 3.20)	0.27 (0.20, 0.37)	0.82 (0.79, 0.85)
NEWS	7	0.75 (0.63, 0.84)	0.65 (0.52, 0.76)	2.13 (1.58, 2.87)	0.39 (0.27, 0.56)	0.76 (0.72, 0.79)
qSOFA	6	0.26 (0.20, 0.33)	0.94 (0.86, 0.97)	4.13 (1.88, 9.08)	0.79 (0.73, 0.86)	0.64 (0.78, 0.84)
**SUBGROUP ANALYSIS**								
**Time of outcome measurement**								
Within 72 h	5	0.88 (0.74, 0.95)	0.56 (0.36, 0.74)	2.01 (1.39, 2.91)	0.21 (0.12, 0.37)	0.82 (0.79, 0.85)
In-hospital	12	0.79 (0.74, 0.84)	0.70 (0.61, 0.77)	2.62 (2.01, 3.42)	0.30 (0.23, 0.39)	0.82 (0.79, 0.85)
**Disease severity**								
Light (mortality rate <10%)	4	0.79 (0.67, 0.87)	0.76 (0.62, 0.86)	3.27 (2.01, 5.32)	0.28 (0.18, 0.45)	0.83 (0.79, 0.86)
Severe (mortality rate ≥10%)	9	0.82 (0.72, 0.89)	0.63 (0.51, 0.47)	2.22 (1.69, 2.92)	0.29 (0.19, 0.43)	0.80 (0.76, 0.83)
**Sensitivity analysis**								
NEWS2 ≥ 5	12	0.82 (0.74, 0.88)	0.66 (0.55, 0.76)	2.44 (1.84, 3.22)	0.27 (0.19, 0.38)	0.82 (0.79, 0.85)
Calculating score at admission	12	0.78 (0.72, 0.83)	0.70 (0.61, 0.77)	2.56 (1.98, 3.32)	0.32 (0.25, 0.41)	0.81 (0.77, 0.84)

*NEWS, National Early Warning Score; NEWS2, National Early Warning Score 2; qSOFA, quick Sequential Organ Failure Assessment; N, number of studies; CI, confidence interval; PLR, positive likelihood ratio; NLR, negative likelihood ratio; AUC, area under the curve*.

### Subgroup and Sensitivity Analyses

There was evidence that the prognostic performance of the NEWS2 varied across different subgroups (Table 4). The performance of the NEWS2 for predicting clinical deterioration within 72 h was better than that during hospitalization (AUC: 0.86 vs. 0.80). In addition, the NEWS2 had more moderate sensitivity and specificity and better discrimination in patients with a less severe disease (mortality rate, <10%).

In sensitivity analyses, we restricted the analyses to studies that evaluated the NEWS2 at hospital admission or studies that used the threshold of ≥5; the pooled sensitivity, specificity, PLR, NLR, and AUC were largely consistent with the primary results (Table 4).

## Discussion

It is vital to determine as quickly as possible which patients with COVID-19 infection are at a high risk of deterioration, especially in poor healthcare resource settings, so as to make proper use of all available resources. To the best of our knowledge, this is the first meta-analysis to evaluate the prognostic accuracy of the NEWS2 on predicting clinical deterioration for patients with COVID-19. In general, the NEWS2 has good discrimination in predicting the combined outcome of the need for intensive respiratory support, admission to the ICU, or in-hospital death. The high sensitivity ensured that the NEWS2 could be used as a sensitive method to initially assess COVID-19 patients at hospital admission. In addition, our results showed that using a threshold of 5 results in high sensitivity (0.83), moderate specificity (0.65), and good discrimination (0.82). It means that early interventions should be implemented for COVID-19 patients with more than five NEWS2 points as soon as possible because the clinical situation of those patients is expected to rapidly deteriorate.

The estimates of the pooled results showed a considerable heterogeneity between studies. Investigating the source of heterogeneity and the prognostic performance of the NEWS2 in different conditions are important objectives in our study. First of all, the NEWS2, an updated version of the NEWS, differs from that of the original NEWS by the inclusion of a new SpO_2_ scoring scale for use in patients with hypercapnic respiratory failure. Oxygen supplementation has been proven to be an independent risk factor for novel coronavirus pneumonia progressing to a critical condition (35). Liu et al. (26) demonstrated that the oxygen saturation level had a good prognostic performance for predicting death in patients with COVID-19 infection. Thus, benefitting from adding a specific scale for patients with hypercapnic respiratory failure, the NEWS2 showed better sensitivity and discrimination than the original NEWS. Second, the time window between score calculation and outcome measurement could also account for heterogeneity. Since predictive accuracy can be improved because the score is calculated close to the occurrence of the outcome, the NEWS2 has a high sensitivity in predicting clinical deterioration within 72 h for patients with COVID-19. The result supports the use of NEWS2 monitoring as a sensitive method to conduct an initial assessment of COVID-19 patients at hospital admission. Third, the severity of a disease might affect the prognostic accuracy as well. For patients with higher mortality rates (≥10%), the NEWS has a high sensitivity but a relatively low specificity, indicating a relatively high false-trigger rate. However, the sensitivity and specificity of the NEWS2 are more moderate in patients with lower mortality (<10%, mostly in the ED). The result supports using the NEWS2 as an adjunct to the process of triage and disposition of newly admitted patients with COVID-19, especially in overcrowded emergency rooms (20). Moreover, study location might be a source of heterogeneity because differences in the healthcare systems of each country could affect clinical outcomes. Specifically, early warning score systems have been introduced and linked to effective clinical responses in many UK hospitals (36). It might introduce the treatment paradox, where some deteriorating patients were likely to receive rapid medical interventions after triggering the alert. Hence, the actual deteriorating rate tends to be lower than predicted and biases our estimate of accuracy. In addition, the primary outcome consists of the need for intensive respiratory support, admission to the ICU, and in-hospital mortality. The indications for the use of intensive respiratory support and the standards of ICU admission were varied among the included studies, which might affect the occurrence of positive results and become a source of heterogeneity.

The NEWS2 is a summary score derived from six physiological parameters; some parameters relate to the degree of respiratory failure, such as oxygen saturation and oxygen supplementation. Since COVID-19 is often characterized by solitary respiratory failure (37, 38), an advantage of NEWS2 compared to other scoring systems is that both hypoxemia and supportive oxygen treatment are included as scoring parameters. It could explain its relatively better performance compared to other scoring systems. In our study, compared with qSOFA, the NEWS had better discrimination and moderate sensitivity and specificity.

Although our research suggests that the NEWS2 has good prognostic performance, it is worth highlighting some potential pitfalls in clinical practice. For instance, in patients with COVID-19, the oxygen requirement might increase rapidly if their respiratory function continued to worsen, but the increased oxygen requirement does not directly cause an increase in the NEWS2 since oxygen supplementation is only a binary variable (yes or no) in the NEWS2 scoring system. Therefore, clinically, we suggest that any increase in oxygen requirement for patients with COVID-19 should arouse the attention of clinicians. Furthermore, given that older patients with COVID-19 have a higher proportion of severe cases and fatality ratio (39, 40), the pandemic has prompted the need to pay particular attention to the health of older persons. Evidence also showed that increased age was independently associated with poor prognosis in COVID-19 patients (8). A Chinese group put forward a modified version of the NEWS2 with the addition of age >65 years as an independent component, termed NEWS-C (41). An external validation study found that the NEWS-C has the best predictive accuracy among common scoring systems for predicting the deterioration of respiratory function in patients with COVID-19 (31). Therefore, it is possible that the prognostic accuracy of the NEWS2 could be improved by modifying the score.

Notably, the NEWS2 is not an alternative to the clinical judgment by experienced clinicians; it should be utilized to help in clinical decision-making by providing objective data. According to the guidelines of the Royal College of Physicians (10), patients with the NEWS2 <5 should also receive strict monitoring because a considerable proportion may still rapidly progress to severe respiratory failure. Finally, in addition to the initial assessment of illness severity, the NEWS was originally designed as a track-and-trigger tool to identify acute clinical deterioration and guide the clinical response for patients. By recording the score on a regular basis, the trends in the clinical response of a patient can be tracked, providing an early warning of clinical deterioration and the need for more intensive treatment (6). Baker et al. found an increasing trend of the NEWS2 beginning many hours prior to the occurrence of a serious clinical deterioration event (18). Therefore, the score should be calculated not only at the admission of patients but also throughout their hospital stay to evaluate a possible deterioration in their clinical situation.

### Strength and Limitations

The strengths of this meta-analysis include, first, a standard protocol and comprehensive search strategies across multiple databases. Thus, we believe that we did not miss any relevant studies. Second, a statistically robust hierarchical model was employed to estimate pooled results and to construct HSROC plots. This approach allows for both between-study variability in sensitivity and specificity and flexibility in the estimation of summary statistics (42). Our findings can contribute to a better understanding of the NEWS2 in patients with COVID-19, which could be useful for implementing the NEWS2 in clinical practice.

Meanwhile, there are some important limitations in the meta-analysis. First, previous research suggest that heterogeneities are widely observed in the systematic reviews of diagnostic test accuracy (43, 44). We also identified significant heterogeneity among the included studies, which might affect the credibility of the pooled estimates. Second, most of the included studies were single-center studies with a relatively small sample size, which may limit the generalizability and certainty of our analysis. Furthermore, the NEWS2 was not designed as a single-time-point predictive tool. Since existing research only show the prognostic accuracy of the NEWS2 in predicting clinical deterioration at a single time point (mostly at the time of admission), we could not evaluate the NEWS2 in any other context. On the other hand, the timings of the NEWS2 measurement were not entirely consistent in the included studies. We assume that the accuracy might be improved if multiple time points were considered, and the changed trend of NEWS2 with time has a potential application value of predicting mortality, just like the delta SOFA (45).

## Conclusion

We perform the first meta-analysis to examine the prognostic accuracy of the NEWS2 on predicting clinical deterioration for patients with COVID-19. The NEWS2 has moderate sensitivity and specificity in predicting the deterioration of patients with COVID-19, and the threshold of 5 is an optimal trigger threshold for activating a rapid response. Our results support the recommendations for use of NEWS2 monitoring as a sensitive method to initially assess COVID-19 patients at hospital admission, although it has a relatively high false-trigger rate. However, the discriminative power of the NEWS2 is far from excellent. Further improvements of the NEWS2 by modifying the score or combining more important predictors is still necessary. In addition, the value of a single assessment is limited. Further research should focus on the utility of longitudinal NEWS2 monitoring to identify deteriorating patients and guide clinical response, not solely for initial assessment at hospital admission.

## Data Availability Statement

The original contributions presented in the study are included in the article/Supplementary Material, further inquiries can be directed to the corresponding author/s.

## Author Contributions

KZ conceived the idea, performed the analysis, and drafted the manuscript. XZ and WD contributed to the study design, data acquisition, and interpretation. NX, BT, and TH helped in the statistical analysis. ZZ and WC critically revised the manuscript for important intellectual content. GZ and HH helped to frame the idea of the study and provided technical support. All authors have read and approved the submitted version.

## Conflict of Interest

The authors declare that the research was conducted in the absence of any commercial or financial relationships that could be construed as a potential conflict of interest.

## Abbreviations

COVID-19: coronavirus disease 2019
SARS-CoV-2: severe acute respiratory syndrome coronavirus 2
UK: United Kingdom
ICU: intensive care unit
ED: emergency department
qSOFA: quick Sequential Organ Failure Assessment
AUC: area under the curve
NEWS: National Early Warning Score
CI: confidence interval
PLR: positive likelihood ratio
NLR: negative likelihood ratio
DOR: diagnostic odds ratio
HSROC: hierarchical summary receiver operating characteristic.

## References

[1] Wang C Horby PW Hayden FG Gao GF. A novel coronavirus outbreak of global health concern. Lancet. (2020) 395:470–3. 10.1016/S0140-6736(20)30185-9 31986257 PMC7135038

[2] WHO Coronavirus (COVID-19) Dashboard. Available online at: https://covid19.who.int/ (accessed March 26, 2021).

[3] Clark A Jit M Warren-Gash C Guthrie B Wang HHX Mercer SW Sanderson C. Global, regional, and national estimates of the population at increased risk of severe COVID-19 due to underlying health conditions in 2020: a modelling study. Lancet Glob Health. (2020) 8:e1003–17. 10.1016/S2214-109X(20)30264-3 32553130 PMC7295519

[4] Wu Z McGoogan JM. Characteristics of and important lessons from the coronavirus disease 2019 (COVID-19) outbreak in China: summary of a report of 72,314 cases from the Chinese Center for Disease Control and Prevention. JAMA. (2020) 323:1239–42. 10.1001/jama.2020.2648 32091533

[5] Farrell TW Ferrante LE Brown T Francis L Widera E Rhodes R. AGS position statement: resource allocation strategies and age-related considerations in the COVID-19 era and beyond. J Am Geriatr Soc. (2020) 68:1136–42. 10.1111/jgs.16537 32374440 PMC7267615

[6] National Early Warning Score (NEWS). Standardising the Assessment of Acute-Illness Severity in the NHS. Report of a working party. London: RCP: Royal College of Physicians (2012).

[7] National Early Warning Score (NEWS) 2. Standardising the Assessment of Acute-Illness Severity in the NHS. Updated report of a working party. London: Royal College of Physicians: RCP (2017).

[8] Zheng Z Peng F Xu B Zhao J Liu H Peng J. Risk factors of critical & mortal COVID-19 cases: a systematic literature review and meta-analysis. J Infect. (2020) 81:e16–25. 10.1016/j.jinf.2020.04.021 PMC7177098 32335169

[9] Zhou F Yu T Du R Fan G Liu Y Liu Z. Clinical course and risk factors for mortality of adult inpatients with COVID-19 in Wuhan, China: a retrospective cohort study. Lancet. (2020) 395:1054–62. 10.1016/S0140-6736(20)30566-3 32171076 PMC7270627

[10] NEWS2 and Deterioration in COVID-19. Available online at: https://www.rcplondon.ac.uk/news/news2-and-deterioration-covid-19 (accessed March 26, 2021).

[11] Swiss Society Of Intensive Care M. Recommendations for the admission of patients with COVID-19 to intensive care and intermediate care units (ICUs and IMCUs). Swiss Med Wkly. (2020) 150:w20227. 10.4414/smw.2020.20227 32208493

[12] Liberati A Altman DG Tetzlaff J Mulrow C Gøtzsche PC Ioannidis JP. The PRISMA statement for reporting systematic reviews and meta-analyses of studies that evaluate healthcare interventions: explanation and elaboration. BMJ. (2009) 339:b2700. 10.1136/bmj.b2700 19622552 PMC2714672

[13] Wolff RF Moons KGM Riley RD Whiting PF Westwood M Collins GS. PROBAST: a tool to assess the risk of bias and applicability of prediction model studies. Ann Intern Med. (2019) 170:51–8. 10.7326/M18-1376 30596875

[14] Reitsma JB Glas AS Rutjes AW Scholten RJ Bossuyt PM Zwinderman AH. Bivariate analysis of sensitivity and specificity produces informative summary measures in diagnostic reviews. J Clin Epidemiol. (2005) 58:982–90. 10.1016/j.jclinepi.2005.02.022 16168343

[15] Higgins JP Thompson SG. Quantifying heterogeneity in a meta-analysis. Stat Med. (2002) 21:1539–58. 10.1002/sim.1186 12111919

[16] Deeks JJ Macaskill P Irwig L. The performance of tests of publication bias and other sample size effects in systematic reviews of diagnostic test accuracy was assessed. J Clin Epidemiol. (2005) 58:882–93. 10.1016/j.jclinepi.2005.01.016 16085191

[17] Aliberti MJR Covinsky KE Garcez FB Smith AK Curiati PK Lee SJ. A fuller picture of COVID-19 prognosis: the added value of vulnerability measures to predict mortality in hospitalised older adults. Age Ageing. (2021) 50:32–9. 10.1093/ageing/afaa240 33068099 PMC7665299

[18] Baker KF Hanrath AT van der Loeff IS Kay LJ Back J Duncan CJ. National early warning score 2 (NEWS2) to identify inpatient COVID-19 deterioration: a retrospective analysis. Clin Med (Lond). (2021) 21:84–9. 10.7861/clinmed.2020-0688 33547065 PMC8002770

[19] Bradley P Frost F Tharmaratnam K Wootton DG. Utility of established prognostic scores in COVID-19 hospital admissions: multicentre prospective evaluation of CURB-65, NEWS2 and qSOFA. BMJ Open Respir Res. (2020) 7:e000729. 10.1136/bmjresp-2020-000729 33293361 PMC7722817

[20] Covino M Sandroni C Santoro M Sabia L Simeoni B Bocci MG. Predicting intensive care unit admission and death for COVID-19 patients in the emergency department using early warning scores. Resuscitation. (2020) 156:84–91. 10.1016/j.resuscitation.2020.08.124 32918985 PMC7480278

[21] Fan G Tu C Zhou F Liu Z Wang Y Song B. Comparison of severity scores for COVID-19 patients with pneumonia: a retrospective study. Eur Respir J. (2020) 56:2002113. 10.1183/13993003.02113-2020 32675205 PMC7366179

[22] Gidari A De Socio GV Sabbatini S Francisci D. Predictive value of national early warning score 2 (NEWS2) for intensive care unit admission in patients with SARS-CoV-2 infection. Infect Dis (Lond). (2020) 52:698–704. 10.1080/23744235.2020.1784457 32584161

[23] Holten AR Nore KG Tveiten C Olasveengen TM Tonby K. Predicting severe COVID-19 in the emergency department. Resusc Plus. (2020) 4:100042. 10.1016/j.resplu.2020.100042 33403367 PMC7577659

[24] Ihle-Hansen H Berge T Tveita A Rønning EJ Ernø PE Andersen EL. COVID-19: symptoms, course of illness and use of clinical scoring systems for the first 42 patients admitted to a Norwegian local hospital. Tidsskr Nor Laegeforen. (2020) 140. 10.4045/tidsskr.20.0301 32378844

[25] Jang JG Hur J Hong KS Lee W Ahn JH. Prognostic accuracy of the SIRS, qSOFA, and NEWS for early detection of clinical deterioration in SARS-CoV-2 infected patients. J Korean Med Sci. (2020) 35:e234. 10.3346/jkms.2020.35.e234 32597046 PMC7324266

[26] Liu FY Sun XL Zhang Y Ge L Wang J Liang X. Evaluation of the risk prediction tools for patients with coronavirus disease 2019 in Wuhan, China: a single-centered, retrospective, observational study. Crit Care Med. (2020) 48:e1004–11. 10.1097/CCM.0000000000004549 32897668 PMC7448719

[27] Maguire D Woods M Richards C Dolan R Veitch JW Sim WMJ. Prognostic factors in patients admitted to an urban teaching hospital with COVID-19 infection. J Transl Med. (2020) 18:354. 10.1186/s12967-020-02524-4 32933530 PMC7491021

[28] Myrstad M Ihle-Hansen H Tveita AA Andersen EL Nygård S Tveit A. National early warning score 2 (NEWS2) on admission predicts severe disease and in-hospital mortality from Covid-19 - a prospective cohort study. Scand J Trauma Resusc Emerg Med. (2020) 28:66. 10.1186/s13049-020-00764-3 32660623 PMC7356106

[29] Pokeerbux MR Yelnik CM Faure E Drumez E Bruandet A Labreuche J. National early warning score to predict intensive care unit transfer and mortality in COVID-19 in a French cohort. Int J Clin Pract. (2021) 75:e14121. 10.1111/ijcp.14121 33650136 PMC7995084

[30] Richardson D Faisal M Fiori M Beatson K Mohammed M. Use of the first National Early Warning Score recorded within 24 hours of admission to estimate the risk of in-hospital mortality in unplanned COVID-19 patients: a retrospective cohort study. BMJ Open. (2021) 11:e043721. 10.1136/bmjopen-2020-043721 33619194 PMC7902318

[31] Su Y Ju MJ Xie RC Yu SJ Zheng JL Ma GG. Prognostic accuracy of early warning scores for clinical deterioration in patients with COVID-19. Front Med (Lausanne). (2021) 7:624255. 10.3389/fmed.2020.624255 33598468 PMC7882600

[32] De Socio GV Gidari A Sicari F Palumbo M Francisci D. National early warning score 2 (NEWS2) better predicts critical coronavirus disease 2019 (COVID-19) illness than COVID-GRAM, a multi-centre study. Infection. (2021) 1–6. 10.1007/s15010-021-01620-x 33970431 PMC8108728

[33] Martin-Rodriguez F Martin-Conty JL Sanz-Garcia A Rodriguez VC Rabbione GO Cebrian Ruiz I. Early warning scores in patients with suspected COVID-19 infection in emergency departments. J Pers Med. (2021) 11:170. 10.3390/jpm11030170 33801375 PMC8001393

[34] Prower E Grant D Bisquera A Breen CP Camporota L Gavrilovski M. The ROX index has greater predictive validity than NEWS2 for deterioration in Covid-19. E Clin Med. (2021) 35:100828. 10.1016/j.eclinm.2021.100828 33937729 PMC8068777

[35] Sun Q Qiu H Huang M Yang Y. Lower mortality of COVID-19 by early recognition and intervention: experience from Jiangsu Province. Ann Intensive Care. (2020) 10:33. 10.1186/s13613-020-00650-2 32189136 PMC7080931

[36] Scott LJ Redmond NM Garrett J Whiting P Northstone K Pullyblank A. Distributions of the national early warning score (NEWS) across a healthcare system following a large-scale roll-out. Emerg Med J. (2019) 36:287–92. 10.1136/emermed-2018-208140 30842204 PMC6580766

[37] Richardson S Hirsch JS Narasimhan M Crawford JM McGinn T Davidson KW. Presenting characteristics, comorbidities, and outcomes among 5700 patients hospitalized with COVID-19 in the New York City Area. JAMA. (2020) 323:2052–9. 10.1001/jama.2020.6775 32320003 PMC7177629

[38] Wu C Chen X Cai Y Xia J Zhou X Xu S. Risk factors associated with acute respiratory distress syndrome and death in patients with coronavirus disease 2019 pneumonia in Wuhan, China. JAMA Intern Med. (2020) 180:934–43. 10.1001/jamainternmed.2020.0994 32167524 PMC7070509

[39] Guan WJ Ni ZY Hu Y Liang WH Ou CQ He JX. Clinical characteristics of coronavirus disease 2019 in China. N Engl J Med. (2020) 382:1708–20. 10.1056/NEJMoa2002032 32109013 PMC7092819

[40] Grasselli G Zangrillo A Zanella A Antonelli M Cabrini L Castelli A. Baseline characteristics and outcomes of 1591 patients infected with SARS-CoV-2 admitted to ICUs of the Lombardy Region, Italy. JAMA. (2020) 323:1574–81. 10.1001/jama.2020.5394 32250385 PMC7136855

[41] Liao X Wang B Kang Y. Novel coronavirus infection during the 2019-2020 epidemic: preparing intensive care units-the experience in Sichuan Province, China. Intensive Care Med. (2020) 46:357–60. 10.1007/s00134-020-05954-2 32025779 PMC7042184

[42] Rutter CM Gatsonis CA. A hierarchical regression approach to meta-analysis of diagnostic test accuracy evaluations. Stat Med. (2001) 20:2865–84. 10.1002/sim.942 11568945

[43] Dinnes J Deeks J Kirby J Roderick P. A methodological review of how heterogeneity has been examined in systematic reviews of diagnostic test accuracy. Health Technol Assess. (2005) 9:1–113, iii. 10.3310/hta9120 15774235

[44] Song JU Sin CK Park HK Shim SR Lee J. Performance of the quick sequential (sepsis-related) organ failure assessment score as a prognostic tool in infected patients outside the intensive care unit: a systematic review and meta-analysis. Critical Care (London, England). (2018) 22:28. 10.1186/s13054-018-1952-x 29409518 PMC5802050

[45] de Grooth HJ Geenen IL Girbes AR Vincent JL Parienti JJ Oudemans-van. SOFA and mortality endpoints in randomized controlled trials: a systematic review and meta-regression analysis. Crit Care (London, England). (2017) 21:38. 10.1186/s13054-017-1609-1 28231816 PMC5324238

