# Supplementary material for: The Prognostic Accuracy of National Early Warning Score 2 on Predicting Clinical Deterioration for Patients With COVID-19: A Systematic Review and Meta-Analysis
Source: Front Med (Lausanne). 2021 Jul 9;8:699880. doi: 10.3389/fmed.2021.699880 (PMC8298908; doi:10.3389/fmed.2021.699880)
Supplement: Supplementary file 2 [file Table_2.PDF]

National Institute for Health Research

UNIVERSITY of York
Centre for Reviews and Dissemination

Systematic review

1. * Review title.

Give the title of the review in English

The prognostic accuracy of Early National Warning Score on predicting for predicting severe disease and in-hospital mortality for Covid-19 patients: a systematic review and meta-analysis

2. Original language title.

For reviews in languages other than English, give the title in the original language. This will be displayed with the English language title.

??

3. * Anticipated or actual start date.

Give the date the systematic review started or is expected to start.

01/03/2021

4. * Anticipated completion date.

Give the date by which the review is expected to be completed.

30/04/2021

5. * Stage of review at time of this submission.

Tick the boxes to show which review tasks have been started and which have been completed. Update this field each time any amendments are made to a published record.

Reviews that have started data extraction (at the time of initial submission) are not eligible for inclusion in PROSPERO. If there is later evidence that incorrect status and/or completion date has been supplied, the published PROSPERO record will be marked as retracted.

This field uses answers to initial screening questions. It cannot be edited until after registration.

The review has not yet started: No

Review stage	Started	Completed
Preliminary searches	Yes	No
Piloting of the study selection process	No	No
Formal screening of search results against eligibility criteria	No	No
Data extraction	No	No
Risk of bias (quality) assessment	No	No
Data analysis	No	No

Provide any other relevant information about the stage of the review here.

6. * Named contact.

The named contact is the guarantor for the accuracy of the information in the register record. This may be any member of the review team.

Kai Zhang

Email salutation (e.g. "Dr Smith" or "Joanne") for correspondence:

Mr. Zhang

7. * Named contact email.

Give the electronic email address of the named contact.

lshyzk@126.com

8. Named contact address

Give the full institutional/organisational postal address for the named contact.

Department of Critical Care Medicine, Second Affiliated Hospital, Zhejiang University School of Medicine, 88 Jiefang Road, Hangzhou, Zhejiang 310009, China

9. Named contact phone number.

Give the telephone number for the named contact, including international dialling code.

15150686177

10. * Organisational affiliation of the review.

Full title of the organisational affiliations for this review and website address if available. This field may be completed as 'None' if the review is not affiliated to any organisation.

Department of Critical Care Medicine, Second Affiliated Hospital, Zhejiang University School of Medicine

Organisation web address:

11. * Review team members and their organisational affiliations.

Give the personal details and the organisational affiliations of each member of the review team. Affiliation refers to groups or organisations to which review team members belong. **NOTE: email and country now MUST be entered for each person, unless you are amending a published record.**

Mr Kai Zhang. Department of Critical Care Medicine, Second Affiliated Hospital, Zhejiang University School of Medicine
Mr Gensheng Zhang. Department of Critical Care Medicine, Second Affiliated Hospital, Zhejiang University School of Medicine

12. * Funding sources/sponsors.

Details of the individuals, organizations, groups, companies or other legal entities who have funded or sponsored the review.

None.

Grant number(s)

State the funder, grant or award number and the date of award

13. * Conflicts of interest.

List actual or perceived conflicts of interest (financial or academic).

None

14. Collaborators.

Give the name and affiliation of any individuals or organisations who are working on the review but who are not listed as review team members. **NOTE: email and country must be completed for each person, unless you are amending a published record.**

15. * Review question.

State the review question(s) clearly and precisely. It may be appropriate to break very broad questions down into a series of related more specific questions. Questions may be framed or refined using PI(E)COS or similar where relevant.

To estimate the prognostic accuracy of Early National Warning Score for predicting severe disease and in-hospital mortality for Covid-19 patients

16. * Searches.

State the sources that will be searched (e.g. Medline). Give the search dates, and any restrictions (e.g. language or publication date). Do NOT enter the full search strategy (it may be provided as a link or attachment below.)

Five electronic databases were searched (PubMed, Embase, Scopus and Cochrane Library) for eligible studies published from December 2019 to March 2021. Full-length articles in English-language journals were eligible.

17. URL to search strategy.

Upload a file with your search strategy, or an example of a search strategy for a specific database, (including the keywords) in pdf or word format. In doing so you are consenting to the file being made publicly accessible. Or provide a URL or link to the strategy. Do NOT provide links to your search **results**.

Alternatively, upload your search strategy to CRD in pdf format. Please note that by doing so you are consenting to the file being made publicly accessible.

Do not make this file publicly available until the review is complete

18. * Condition or domain being studied.

Give a short description of the disease, condition or healthcare domain being studied in your systematic review.

The rapid spread of the severe acute respiratory syndrome coronavirus 2 (SARS-CoV-2) has burdened most healthcare systems worldwide. Although most individuals with coronavirus disease 2019 (COVID-19) are asymptomatic or oligosymptomatic, one in five persons develop severe forms of the disease. In this context, front line health workers are constantly challenged to determine the severity and prognosis of COVID-19 cases to provide high-quality care and efficiently allocate resources. In past decades, various screening tools have been incorporated into the triage systems to identify patients at a higher risk of clinical deterioration in acute care settings. These instruments use physiological parameters, such as vital signs, to measure illness acuity (general level of patient illness, urgency for clinical intervention and intensity of resource utilisation). Previous studies have shown that tools such as the National Early Warning Score (NEWS) can be used to identify patients with infection who are at a higher risk of mortality in the hospital.

19. * Participants/population.

Specify the participants or populations being studied in the review. The preferred format includes details of both inclusion and exclusion criteria.

Adult patients (aged ?18 years old) with confirmed (detection of the new coronavirus using reverse transcription polymerase chain reactions) cases of SARS-CoV-2 infection.

20. * Intervention(s), exposure(s).

Give full and clear descriptions or definitions of the interventions or the exposures to be reviewed. The preferred format includes details of both inclusion and exclusion criteria.

National Early Warning Score measurement done during the hospitalization to predict the severe disease and in-hospital mortality.

21. * Comparator(s)/control.

Where relevant, give details of the alternatives against which the intervention/exposure will be compared (e.g. another intervention or a non-exposed control group). The preferred format includes details of both inclusion and exclusion criteria.

Not applicable

22. * Types of study to be included.

Give details of the study designs (e.g. RCT) that are eligible for inclusion in the review. The preferred format includes both inclusion and exclusion criteria. If there are no restrictions on the types of study, this should be stated.

Observational studies

23. Context.

Give summary details of the setting or other relevant characteristics, which help define the inclusion or exclusion criteria.

24. * Main outcome(s).

Give the pre-specified main (most important) outcomes of the review, including details of how the outcome is defined and measured and when these measurement are made, if these are part of the review inclusion criteria.

Severe or mortal COVID-19 cases

Measures of effect

Please specify the effect measure(s) for you main outcome(s) e.g. relative risks, odds ratios, risk difference, and/or 'number needed to treat.

Severe COVID-19 cases including patients need respiratory support, admission to intensive care unit.

Mortality events including all-cause mortality in hospital.

25. * Additional outcome(s).

List the pre-specified additional outcomes of the review, with a similar level of detail to that required for main outcomes. Where there are no additional outcomes please state 'None' or 'Not applicable' as appropriate to the review

None.

Measures of effect

Please specify the effect measure(s) for you additional outcome(s) e.g. relative risks, odds ratios, risk difference, and/or 'number needed to treat.

Not applicable.

26. * Data extraction (selection and coding).

Describe how studies will be selected for inclusion. State what data will be extracted or obtained. State how this will be done and recorded.

One investigator independently collected the following variables from the included articles: author information, year of publication, country, study design, number of patients, mean or median age, sex ratio and mortality (in-hospital, 28-day, or 30-day). Two investigators independently collected true-positive, false-positive, true-negative, and false-negative counts; the total number of survivors and cases; and sensitivity, specificity, AUROC of National Early Warning Score. Disagreements were resolved by consensus.

27. * Risk of bias (quality) assessment.

State which characteristics of the studies will be assessed and/or any formal risk of bias/quality assessment tools that will be used.

PROBAST (prediction model risk of bias assessment tool) was used to assess the quality.

28. * Strategy for data synthesis.

Describe the methods you plan to use to synthesise data. This **must not be generic text** but should be **specific to your review** and describe how the proposed approach will be applied to your data. If meta-

analysis is planned, describe the models to be used, methods to explore statistical heterogeneity, and software package to be used.

The data will be presented as mean values for continuous variables and as frequencies (%) for categorical variables. For the diagnostic meta-analysis, we will extract the number of patients with true-positive, false-positive, false-negative, and true-negative test results either directly, or through a recalculation based on the reported measures of accuracy in combination with the prevalence and sample sizes from the included studies. We will then calculate the pooled sensitivity and specificity, positive likelihood ratio (PLR), negative likelihood ratio (NLR), diagnostic odds ratios (DORs) as point estimates with 95% confidence intervals (CI), and will also construct hierarchical summary receiver operating characteristics (HSROC) curves to overcome some limitations of the traditional summary ROC curve procedure. Between-study statistical heterogeneity will be assessed using I^2 and Cochran's Q test, with I^2 values 50% indicating substantial levels of between-study heterogeneity, requiring the use of a random-effects model otherwise (for values 50%, a fixed-effect model will be used). In addition, if there is found to be a substantial level of heterogeneity, analysis via meta-regression will be performed to identify potential sources of bias. Publication bias will also be evaluated using the Deek test for funnel plot asymmetry, and a P value 0.05 will be considered as statistically significant. All analyses will be performed using Revman 5.3 and Stata 14.0.

29. * Analysis of subgroups or subsets.

State any planned investigation of ‘subgroups’. Be clear and specific about which type of study or participant will be included in each group or covariate investigated. State the planned analytic approach.

Subgroup analyses were planned to further investigate heterogeneity of studies by version of NEWS (NEWS versus NEWS2), time of outcome measurement (short-term mortality versus in-hospital mortality), disease severity (mortality rate 10% versus ?10%), outcome definition (severe COVID-19 versus mortality). Sensitivity analyses were also conducted by repeating the analyses within studies used the threshold value of ?5.

30. * Type and method of review.

Select the type of review, review method and health area from the lists below.

Type of review

Cost effectiveness
No

Diagnostic
No

Epidemiologic
No

Individual patient data (IPD) meta-analysis
No

Intervention
No

Living systematic review
No

Meta-analysis
Yes

Methodology
No

Narrative synthesis
No

Network meta-analysis
No

Pre-clinical
No

Prevention
No

Prognostic
No

Prospective meta-analysis (PMA)
No

Review of reviews
No

Service delivery
No

Synthesis of qualitative studies
No

Systematic review
Yes

Other
No

Health area of the review

Alcohol/substance misuse/abuse
No

Blood and immune system
No

Cancer
No

Cardiovascular
No

Care of the elderly
No

Child health
No

Complementary therapies

No

COVID-19
Yes

For COVID-19 registrations please tick all categories that apply. Doing so will enable your record to appear in area-specific searches

Chinese medicine
Diagnosis
Epidemiological
Genetics
Health impacts
Immunity
Long COVID
Mental health
PPE
Prognosis
Public health intervention
Rehabilitation
Service delivery
Transmission
Treatments
Vaccines
Other

Crime and justice
No

Dental
No

Digestive system
No

Ear, nose and throat
No

Education
No

Endocrine and metabolic disorders
No

Eye disorders
No

General interest
No

Genetics
No

Health inequalities/health equity
No

Infections and infestations
Yes

International development
No

Mental health and behavioural conditions

No

Musculoskeletal
No

Neurological
No

Nursing
No

Obstetrics and gynaecology
No

Oral health
No

Palliative care
No

Perioperative care
No

Physiotherapy
No

Pregnancy and childbirth
No

Public health (including social determinants of health)
No

Rehabilitation
No

Respiratory disorders
Yes

Service delivery
No

Skin disorders
No

Social care
No

Surgery
No

Tropical Medicine
No

Urological
No

Wounds, injuries and accidents
No

Violence and abuse
No

31. Language.

Select each language individually to add it to the list below, use the bin icon to remove any added in error.

English

There is not an English language summary

32. * Country.

Select the country in which the review is being carried out. For multi-national collaborations select all the countries involved.

China

33. Other registration details.

Name any other organisation where the systematic review title or protocol is registered (e.g. Campbell, or The Joanna Briggs Institute) together with any unique identification number assigned by them. If extracted data will be stored and made available through a repository such as the Systematic Review Data Repository (SRDR), details and a link should be included here. If none, leave blank.

34. Reference and/or URL for published protocol.

If the protocol for this review is published provide details (authors, title and journal details, preferably in Vancouver format)

Add web link to the published protocol.

Or, upload your published protocol here in pdf format. Note that the upload will be publicly accessible.

No I do not make this file publicly available until the review is complete

Please note that the information required in the PROSPERO registration form must be completed in full even if access to a protocol is given.

35. Dissemination plans.

Do you intend to publish the review on completion?

No

Give brief details of plans for communicating review findings.?

36. Keywords.

Give words or phrases that best describe the review. Separate keywords with a semicolon or new line. Keywords help PROSPERO users find your review (keywords do not appear in the public record but are included in searches). Be as specific and precise as possible. Avoid acronyms and abbreviations unless these are in wide use.

37. Details of any existing review of the same topic by the same authors.

If you are registering an update of an existing review give details of the earlier versions and include a full bibliographic reference, if available.

38. * Current review status.

Update review status when the review is completed and when it is published.New registrations must be ongoing so this field is not editable for initial submission.

Please provide anticipated publication date

Review_Ongoing

39. Any additional information.

Provide any other information relevant to the registration of this review.

40. Details of final report/publication(s) or preprints if available.

Leave empty until publication details are available OR you have a link to a preprint (NOTE: this field is not editable for initial submission). List authors, title and journal details preferably in Vancouver format.

Give the link to the published review or preprint.

In the meta-analysis, there are some deviations from the PROSPERO protocol:

1. In the protocol, the main outcome was severe or mortal COVID-19 cases. In the manuscript, we combined the severe and mortal COVID-19 cases as clinical deterioration (including the need for intensive respiratory support, admission to ICU, or in-hospital death).

2. Since the updated NEWS2 was recommended for initial assessment in patients with COVID-19, we focused on the prognostic accuracy of NEWS2 rather than the original NEWS. In addition, we compared the prognostic accuracy of NEWS2 with the original NEWS and qSOFA.

3. In the subgroup analyses, we deleted the analyses of the outcome definition (because most of included studies reported a composite outcome including severe and mortal COVID-19 cases).

4. In the sensitivity analyses, we added supplemental statistics by repeating the analyses within studies evaluated the NEWS2 at hospital admission.
