# Supplementary material for: The Prognostic Accuracy of National Early Warning Score 2 on Predicting Clinical Deterioration for Patients With COVID-19: A Systematic Review and Meta-Analysis
Source: Front Med (Lausanne). 2021 Jul 9;8:699880. doi: 10.3389/fmed.2021.699880 (PMC8298908; doi:10.3389/fmed.2021.699880)
Supplement: Supplementary file 3 [file Table_3.DOCX]

**Supplementary Material 3** **Searching strategies,** **inclusion and exclusion criteria, and quality assessment criteria**

**Searching strategies**

**Pubmed**

#1 novel coronavirus [Title/Abstract] OR COVID [Title/Abstract] OR COVID-19 [Title/Abstract] OR new coronavirus [Title/Abstract] OR SARS CoV-2 [Title/Abstract] OR SARS-CoV-2 [Title/Abstract] OR nCoV [Title/Abstract]

#2 national early warning score [Title/Abstract] OR prognostic [Title/Abstract] OR predictive [Title/Abstract]

#1 AND #2

**Embase**

#1 ‘novel coronavirus’:ab,ti OR ‘novel COVID’:ab,ti OR ‘new coronavirus’:ab,ti OR ‘SARS CoV-2’:ab,ti OR ‘SARS-CoV-2’:ab,ti OR ‘COVID’:ab,ti

#2 (national early warning score):ab,ti OR ‘prognostic’:ab,ti OR ‘predictive’:ab,ti

#1 AND #2

**Scopus**

#1 TITLE-ABS-KEY (novel coronavirus) OR TITLE-ABS-KEY (novel COVID) OR TITLE-ABS-KEY (new coronavirus) OR TITLE-ABS-KEY (SARS CoV-2) OR TITLE-ABS-KEY (*nCoV*) OR TITLE-ABS-KEY (COVID) OR TITLE-ABS-KEY (SARS-CoV-2)

#2 TITLE-ABS-KEY (national early warning score) OR TITLE-ABS-KEY (prognostic) OR TITLE-ABS-KEY (predictive)

#1 AND #2

**Cochrane Library**

#1 ‘novel coronavirus’:ti,ab,kw OR ‘novel COVID’: ti,ab,kw OR ‘new coronavirus’: ti,ab,kw OR ‘SARS CoV-2’: ti,ab,kw OR ‘SARS-CoV-2’: ti,ab,kw OR ‘COVID’: ti,ab,kw

#2 (national early warning score): ti,ab,kw OR ‘prognostic’: ti,ab,kw OR ‘predictive’: ti,ab,kw

#1 AND #2

**Inclusion criteria**

(1) The study recruited adult patients (≥18 years old) with confirmed cases of SARS-CoV-2 infection;

(2) The study applied the NEWS2 or NEWS for predicting clinical deterioration (including need for intensive respiratory support, admission to ICU, or in-hospital death);

(3) The study should provide sufficient data to calculate the true positive (TP), false positive (FP), false negative (FN), true negative (TN) results;

(4) Full-length articles and the research objects are limited to human;

**Exclusion criteria**

1. The study population included non-adult or non-COVID-19 patients;
2. The study did not report sufficient data to calculate TP, FP, FN, and TN results;
3. Case reports, animal studies, pediatric studies;
4. If studies used the same database, we included the study with the most patients and excluded the others

**Quality Assessment Criteria**

Two authors independently assessed the risk of bias of included trials by using the Prediction model Risk Of Bias ASsessment Tool (PROBAST). The PROBAST consists assessment of four key domains to judge the quality of studies: participants, predictors, outcome, analysis. The answer to each item was “+”, “-” or “?” (“+” indicates low risk of bias; “-” indicates high risk of bias; and “?” indicates unclear risk of bias). If a study was judged as “low” on all domains relating to bias, then it was assigned an overall judgment of “low risk of bias” or “low concern regarding applicability”, and had high quality. If a study was judged “high” in one or more domains, then it may have been judged as “at risk of bias” or “concerns regarding applicability”. Disagreements were resolved by third author.
