# Supplementary material for: The Prognostic Accuracy of National Early Warning Score 2 on Predicting Clinical Deterioration for Patients With COVID-19: A Systematic Review and Meta-Analysis
Source: Front Med (Lausanne). 2021 Jul 9;8:699880. doi: 10.3389/fmed.2021.699880 (PMC8298908; doi:10.3389/fmed.2021.699880)
Supplement: Supplementary file 4 [file Table_4.DOCX]

**Supplementary File 4 Excluded studies with reasons**

| **Study** | **Reason** |
| --- | --- |
| Carr[1] | Modified NEWS |
| Covino[2] | The study used the same cohort of patients with an already included study |
| Vernaz[3] | Intervention study |
| Hu[4] | Improper threshold value (NEWS2≥10) |
| Hong[5] | No concerned outcome or the relevant data not described |
| Kostakis[6] | No concerned outcome or the relevant data not described |
| Mitacchione[7] | Intervention study |
| Schneider[8] | Intervention study |
| Galassi[9] | Modified NEWS |
| Raina[10] | Non-adult |
| Gupta-Wright[11] | Modified NEWS |
| Arnold[12] | Improper threshold value (the threshold value was not reported) |
| Fox[13] | Only abstract |
| de Roos[14] | No concerned outcome or the relevant data not described |
| Carallo[15] | No concerned outcome or the relevant data not described |
| Hu[16] | No concerned outcome or the relevant data not described |
| Johnston[17] | Only abstract |
| Garazzino[18] | Non-adult |
| Motta[19] | No concerned outcome or the relevant data not described |
| Leger[20] | No concerned outcome or the relevant data not described |
| Un[21] | No concerned outcome or the relevant data not described |
| Madsen[22] | No concerned outcome or the relevant data not described |

**Reference**

1. Carr E, Bendayan R, Bean D, Stammers M, Wang W, Zhang H, Searle T, Kraljevic Z, Shek A, Phan HTT *et al*: **Evaluation and improvement of the National Early Warning Score (NEWS2) for COVID-19: a multi-hospital study**. *BMC Med* 2021, **19**(1):23.

2. Covino M, De Matteis G, Burzo ML, Russo A, Forte E, Carnicelli A, Piccioni A, Simeoni B, Gasbarrini A, Franceschi F *et al*: **Predicting In-Hospital Mortality in COVID-19 Older Patients with Specifically Developed Scores**. *J Am Geriatr Soc* 2021, **69**(1):37-43.

3. Vernaz N, Agoritsas T, Calmy A, Gayet-Ageron A, Gold G, Perrier A, Picard F, Prendki V, Reny JL, Samer C *et al*: **Early experimental COVID-19 therapies: associations with length of hospital stay, mortality and related costs**. *Swiss Med Wkly* 2020, **150**:w20446.

4. Hu H, Yao N, Qiu Y: **Predictive Value of 5 Early Warning Scores for Critical COVID-19 Patients**. *Disaster medicine and public health preparedness* 2020:1-8.

5. Hong KS, Lee KH, Chung JH, Shin KC, Choi EY, Jin HJ, Jang JG, Lee W, Ahn JH: **Clinical Features and Outcomes of 98 Patients Hospitalized with SARS-CoV-2 Infection in Daegu, South Korea: A Brief Descriptive Study**. *Yonsei Med J* 2020, **61**(5):431-437.

6. Kostakis I, Smith GB, Prytherch D, Meredith P, Price C, Chauhan A: **The performance of the National Early Warning Score and National Early Warning Score 2 in hospitalised patients infected by the severe acute respiratory syndrome coronavirus 2 (SARS-CoV-2)**. *Resuscitation* 2021, **159**:150-157.

7. Mitacchione G, Schiavone M, Curnis A, Arca M, Antinori S, Gasperetti A, Mascioli G, Severino P, Sabato F, Caracciolo MM *et al*: **Impact of prior statin use on clinical outcomes in COVID-19 patients: data from tertiary referral hospitals during COVID-19 pandemic in Italy**. *J Clin Lipidol* 2021, **15**(1):68-78.

8. Schneider J, Jaenigen B, Wagner D, Rieg S, Hornuss D, Biever PM, Kern WV, Walz G: **Therapy with lopinavir/ritonavir and hydroxychloroquine is associated with acute kidney injury in COVID-19 patients**. *PLoS One* 2021, **16**(5):e0249760.

9. Galassi L, Schena D: **The Modified National Early Warning Score (m-NEWS) for COVID-19-Infected Patient Evaluation: a Proof-of-Concept**. *SN Compr Clin Med* 2021:1-2.

10. Raina R, Chakraborty R, Mawby I, Agarwal N, Sethi S, Forbes M: **Critical analysis of acute kidney injury in pediatric COVID-19 patients in the intensive care unit**. *Pediatr Nephrol* 2021:1-12.

11. Gupta-Wright A, Macleod CK, Barrett J, Filson SA, Corrah T, Parris V, Sandhu G, Harris M, Tennant R, Vaid N *et al*: **False-negative RT-PCR for COVID-19 and a diagnostic risk score: a retrospective cohort study among patients admitted to hospital**. *BMJ Open* 2021, **11**(2):e047110.

12. Arnold DT, Attwood M, Barratt S, Morley A, Elvers KT, McKernon J, Donald C, Oates A, Noel A, MacGowan A *et al*: **Predicting outcomes of COVID-19 from admission biomarkers: a prospective UK cohort study**. *Emerg Med J* 2021.

13. Fox L, Kostakis I, Price C, Smith G, Prytherch D, Meredith P, Chauhan A: **The performance of the national early warning score and national early warning score 2 in hospitalised patients infected by the severe acute respiratory syndrome coronavirus 2 (SARS-COV-2)**. *Thorax* 2021, **76**(SUPPL 1):A59.

14. de Roos MP, Kilsdonk ID, Hekking PW, Peringa J, Dijkstra NG, Kunst PWA, Bresser P, Reesink HJ: **Chest computed tomography and alveolar-arterial oxygen gradient as rapid tools to diagnose and triage mildly symptomatic COVID-19 pneumonia patients**. *ERJ Open Res* 2021, **7**(1).

15. Carallo C, Pugliese F, Tripolino C, Lenzi L, Oliveri C, Fasani G, Guarrera GM, Spagnolli W, Cozzio S: **Early-stage predictors of the acute phase duration in uncomplicated COVID-19 pneumonia**. *J Med Virol* 2021, **93**(1):513-517.

16. Hu H, Yao N, Qiu Y: **Predictive Value of 5 Early Warning Scores for Critical COVID-19 Patients**. *Disaster Med Public Health Prep* 2020:1-8.

17. Johnston BW, Toal C, Morgan E, Mulla S, Ross A, Sexton N, Spiliopoulos M, Thompson W, Waite S, White S *et al*: **An observational cohort study of physiological parameters that predict the need for mechanical ventilation and intensive care unit admission in patients admitted to hospital with due to SARS-CoV-2 infection**. *Intensive Care Medicine Experimental* 2020, **8**(SUPPL 2).

18. Garazzino S, Lo Vecchio A, Pierantoni L, Calò Carducci FI, Marchetti F, Meini A, Castagnola E, Vergine G, Donà D, Bosis S *et al*: **Epidemiology, Clinical Features and Prognostic Factors of Pediatric SARS-CoV-2 Infection: Results From an Italian Multicenter Study**. *Front Pediatr* 2021, **9**:649358.

19. Motta JC, Novoa DJ, Gómez CC, Moreno JM, Vargas L, Pérez J, Millán H, Arango Á I: **Prognostic factors in hospitalized patients diagnosed with SARS-CoV-2 infection, Bogotá, Colombia**. *Biomedica* 2020, **40**(Supl. 2):116-130.

20. Leger T, Jacquier A, Barral PA, Castelli M, Finance J, Lagier JC, Million M, Parola P, Brouqui P, Raoult D *et al*: **Low-dose chest CT for diagnosing and assessing the extent of lung involvement of SARS-CoV-2 pneumonia using a semi quantitative score**. *PLoS One* 2020, **15**(11):e0241407.

21. Un KC, Wong CK, Lau YM, Lee JC, Tam FC, Lai WH, Lau YM, Chen H, Wibowo S, Zhang X *et al*: **Observational study on wearable biosensors and machine learning-based remote monitoring of COVID-19 patients**. *Sci Rep* 2021, **11**(1):4388.

22. Madsen LW, Lindvig SO, Rasmussen LD, Knudtzen FC, Laursen CB, Øvrehus A, Nielsen SL, Johansen IS: **Low mortality of hospitalised patients with COVID-19 in a tertiary Danish hospital setting**. *Int J Infect Dis* 2021, **102**:212-219.
