# Supplementary material for: The Prognostic Accuracy of National Early Warning Score 2 on Predicting Clinical Deterioration for Patients With COVID-19: A Systematic Review and Meta-Analysis
Source: Front Med (Lausanne). 2021 Jul 9;8:699880. doi: 10.3389/fmed.2021.699880 (PMC8298908; doi:10.3389/fmed.2021.699880)
Supplement: Supplementary file 5 [file Table_5.PDF]

Aliberiti et al.

DOMAIN 1: Participants		
**A. Risk of Bias**		
1.1 Were appropriate data sources used, e.g. cohort, RCT or nested case-control study data?		Yes
1.2 Were all inclusions and exclusions of participants appropriate?		No
**Risk of bias introduced by selection of participants**	**RISK:** (low/ high/ unclear)	High
Rationale of bias rating: Only patients aged ≥50 years were included		
**B. Applicability**		
**Concern that the included participants and setting do not match the review question**	**CONCERN:** (low/ high/ unclear)	Low
Rationale of applicability rating: Match the review question		

DOMAIN 2: Predictors		
**A. Risk of Bias**		
2.1 Were predictors defined and assessed in a similar way for all participants?		Yes
2.2 Were predictor assessments made without knowledge of outcome data?		Yes
2.3 Are all predictors available at the time the model is intended to be used?		Yes
**Risk of bias introduced by predictors or their assessment**	**RISK:** (low/ high/ unclear)	Low
Rationale of bias rating: Low risk of bias		
**B. Applicability**		
Concern that the definition, assessment or timing of predictors in the model do not match the review question	**CONCERN:** (low/ high/ unclear)	High
Rationale of applicability rating: The threshold value of NEWS was not consistent with other studies		

DOMAIN 3: Outcome		
**A. Risk of Bias**		
3.1 Was the outcome determined appropriately?		Yes
3.2 Was a pre-specified or standard outcome definition used?		Yes
3.3 Were predictors excluded from the outcome definition?		Yes
3.4 Was the outcome defined and determined in a similar way for all participants?		Yes
3.5 Was the outcome determined without knowledge of predictor information?		Yes
3.6 Was the time interval between predictor assessment and outcome determination appropriate?		Yes
**Risk of bias introduced by the outcome or its determination**	**RISK:** (low/ high/ unclear)	Low
Rationale of bias rating: Low risk of bias		
**B. Applicability**		
**Concern that the outcome, its definition, timing or determination do not match the review question**	**CONCERN:** (low/ high/ unclear)	Low
Rationale of applicability rating: Match the review question		

DOMAIN 4: Analysis		
**Risk of Bias**		
4.1 Were there a reasonable number of participants with the outcome?		Yes
4.2 Were continuous and categorical predictors handled appropriately?		Yes
4.3 Were all enrolled participants included in the analysis?		Yes
4.4 Were participants with missing data handled appropriately?		Yes
4.5 Was selection of predictors based on univariable analysis avoided?		Not applicable
4.6 Were complexities in the data (e.g. censoring, competing risks, sampling of controls) accounted for appropriately?		Yes
4.7 Were relevant model performance measures evaluated appropriately?		No information
4.8 Were model overfitting and optimism in model performance accounted for?		Not applicable
4.9 Do predictors and their assigned weights in the final model correspond to the results from multivariable analysis?		Not applicable
**Risk of bias introduced by the analysis**	**RISK:** (low/ high/ unclear)	Unclear
Rationale of bias rating: Did not report the calibration of the NEWS		

Baker et al.

DOMAIN 1: Participants		
**A. Risk of Bias**		
1.1 Were appropriate data sources used, e.g. cohort, RCT or nested case-control study data?		Yes
1.2 Were all inclusions and exclusions of participants appropriate?		Yes
**Risk of bias introduced by selection of participants**	**RISK:** (low/ high/ unclear)	Low
Rationale of bias rating: Low risk of bias		
**B. Applicability**		
**Concern that the included participants and setting do not match the review question**	**CONCERN:** (low/ high/ unclear)	Low
Rationale of applicability rating: Match the review question		

DOMAIN 2: Predictors		
**A. Risk of Bias**		
2.1 Were predictors defined and assessed in a similar way for all participants?		Yes
2.2 Were predictor assessments made without knowledge of outcome data?		Yes
2.3 Are all predictors available at the time the model is intended to be used?		Yes
**Risk of bias introduced by predictors or their assessment**	**RISK:** (low/ high/ unclear)	Low
Rationale of bias rating: Low risk of bias		
**B. Applicability**		
Concern that the definition, assessment or timing of predictors in the model do not match the review question	**CONCERN:** (low/ high/ unclear)	Low
Rationale of applicability rating: Match the review question		

DOMAIN 3: Outcome		
**A. Risk of Bias**		
3.1 Was the outcome determined appropriately?		Yes
3.2 Was a pre-specified or standard outcome definition used?		Yes
3.3 Were predictors excluded from the outcome definition?		Yes
3.4 Was the outcome defined and determined in a similar way for all participants?		Yes
3.5 Was the outcome determined without knowledge of predictor information?		Yes
3.6 Was the time interval between predictor assessment and outcome determination appropriate?		No
**Risk of bias introduced by the outcome or its determination**	**RISK:** (low/ high/ unclear)	High
Rationale of bias rating: The time interval between predictor assessment and outcome determination was too short		
**B. Applicability**		
**Concern that the outcome, its definition, timing or determination do not match the review question**	**CONCERN:** (low/ high/ unclear)	Low
Rationale of applicability rating: Match the review question		

DOMAIN 4: Analysis		
**Risk of Bias**		
4.1 Were there a reasonable number of participants with the outcome?		Yes
4.2 Were continuous and categorical predictors handled appropriately?		Yes
4.3 Were all enrolled participants included in the analysis?		Yes
4.4 Were participants with missing data handled appropriately?		No
4.5 Was selection of predictors based on univariable analysis avoided?		Not applicable
4.6 Were complexities in the data (e.g. censoring, competing risks, sampling of controls) accounted for appropriately?		Yes
4.7 Were relevant model performance measures evaluated appropriately?		No information
4.8 Were model overfitting and optimism in model performance accounted for?		Not applicable
4.9 Do predictors and their assigned weights in the final model correspond to the results from multivariable analysis?		Not applicable
**Risk of bias introduced by the analysis**	**RISK:** (low/ high/ unclear)	High
Rationale of bias rating: Patients with missing data were excluded		

Bradley et al.

DOMAIN 1: Participants		
**A. Risk of Bias**		
1.1 Were appropriate data sources used, e.g. cohort, RCT or nested case-control study data?		Yes
1.2 Were all inclusions and exclusions of participants appropriate?		Yes
**Risk of bias introduced by selection of participants**	**RISK:** (low/ high/ unclear)	Low
Rationale of bias rating:<br>Low risk of bias		
**B. Applicability**		
**Concern that the included participants and setting do not match the review question**	**CONCERN:** (low/ high/ unclear)	Low
Rationale of applicability rating:<br>Match the review question		

DOMAIN 2: Predictors		
**A. Risk of Bias**		
2.1 Were predictors defined and assessed in a similar way for all participants?		Yes
2.2 Were predictor assessments made without knowledge of outcome data?		Yes
2.3 Are all predictors available at the time the model is intended to be used?		Yes
**Risk of bias introduced by predictors or their assessment**	**RISK:** (low/ high/ unclear)	Low
Rationale of bias rating:<br>Low risk of bias		
**B. Applicability**		
Concern that the definition, assessment or timing of predictors in the model do not match the review question	**CONCERN:** (low/ high/ unclear)	Low
Rationale of applicability rating:<br>Match the review question		

DOMAIN 3: Outcome		
**A. Risk of Bias**		
3.1 Was the outcome determined appropriately?		Yes
3.2 Was a pre-specified or standard outcome definition used?		Yes
3.3 Were predictors excluded from the outcome definition?		Yes
3.4 Was the outcome defined and determined in a similar way for all participants?		Yes
3.5 Was the outcome determined without knowledge of predictor information?		Yes
3.6 Was the time interval between predictor assessment and outcome determination appropriate?		No information
**Risk of bias introduced by the outcome or its determination**	**RISK:** (low/ high/ unclear)	Unclear
Rationale of bias rating:<br>The time interval between predictor assessment and outcome determination was not specified		
**B. Applicability**		
**Concern that the outcome, its definition, timing or determination do not match the review question**	**CONCERN:** (low/ high/ unclear)	Unclear
Rationale of applicability rating:<br>The time interval between predictor assessment and outcome determination was not specified		

DOMAIN 4: Analysis		
**Risk of Bias**		
4.1 Were there a reasonable number of participants with the outcome?		Yes
4.2 Were continuous and categorical predictors handled appropriately?		Yes
4.3 Were all enrolled participants included in the analysis?		Yes
4.4 Were participants with missing data handled appropriately?		No
4.5 Was selection of predictors based on univariable analysis avoided?		Not applicable
4.6 Were complexities in the data (e.g. censoring, competing risks, sampling of controls) accounted for appropriately?		Yes
4.7 Were relevant model performance measures evaluated appropriately?		Yes
4.8 Were model overfitting and optimism in model performance accounted for?		Not applicable
4.9 Do predictors and their assigned weights in the final model correspond to the results from multivariable analysis?		Not applicable
**Risk of bias introduced by the analysis**	**RISK:** (low/ high/ unclear)	High
Rationale of bias rating: Patients with missing data were excluded		

Covino et al.

DOMAIN 1: Participants		
**A. Risk of Bias**		
1.1 Were appropriate data sources used, e.g. cohort, RCT or nested case-control study data?		Yes
1.2 Were all inclusions and exclusions of participants appropriate?		Yes
**Risk of bias introduced by selection of participants**	**RISK:** (low/ high/ unclear)	Low
Rationale of bias rating: Low risk of bias		
**B. Applicability**		
**Concern that the included participants and setting do not match the review question**	**CONCERN:** (low/ high/ unclear)	Low
Rationale of applicability rating: Match the review question		

DOMAIN 2: Predictors		
**A. Risk of Bias**		
2.1 Were predictors defined and assessed in a similar way for all participants?		Yes
2.2 Were predictor assessments made without knowledge of outcome data?		Yes
2.3 Are all predictors available at the time the model is intended to be used?		Yes
**Risk of bias introduced by predictors or their assessment**	**RISK:** (low/ high/ unclear)	Low
Rationale of bias rating: Low risk of bias		
**B. Applicability**		
Concern that the definition, assessment or timing of predictors in the model do not match the review question	**CONCERN:** (low/ high/ unclear)	Low
Rationale of applicability rating: Match the review question		

DOMAIN 3: Outcome		
**A. Risk of Bias**		
3.1 Was the outcome determined appropriately?		Yes
3.2 Was a pre-specified or standard outcome definition used?		Yes
3.3 Were predictors excluded from the outcome definition?		Yes
3.4 Was the outcome defined and determined in a similar way for all participants?		Yes
3.5 Was the outcome determined without knowledge of predictor information?		Yes
3.6 Was the time interval between predictor assessment and outcome determination appropriate?		Yes
**Risk of bias introduced by the outcome or its determination**	**RISK:** (low/ high/ unclear)	Low
Rationale of bias rating: Low risk of bias		
**B. Applicability**		
**Concern that the outcome, its definition, timing or determination do not match the review question**	**CONCERN:** (low/ high/ unclear)	Low
Rationale of applicability rating: Match the review question		

DOMAIN 4: Analysis		
**Risk of Bias**		
4.1 Were there a reasonable number of participants with the outcome?		Yes
4.2 Were continuous and categorical predictors handled appropriately?		Yes
4.3 Were all enrolled participants included in the analysis?		Yes
4.4 Were participants with missing data handled appropriately?		No
4.5 Was selection of predictors based on univariable analysis avoided?		Not applicable
4.6 Were complexities in the data (e.g. censoring, competing risks, sampling of controls) accounted for appropriately?		Yes
4.7 Were relevant model performance measures evaluated appropriately?		No information
4.8 Were model overfitting and optimism in model performance accounted for?		Not applicable
4.9 Do predictors and their assigned weights in the final model correspond to the results from multivariable analysis?		Not applicable
**Risk of bias introduced by the analysis**	**RISK:** (low/ high/ unclear)	High
Rationale of bias rating: Patients with missing data were excluded		

De Socio et al.

DOMAIN 1: Participants		
**A. Risk of Bias**		
1.1 Were appropriate data sources used, e.g. cohort, RCT or nested case-control study data?		Yes
1.2 Were all inclusions and exclusions of participants appropriate?		Yes
**Risk of bias introduced by selection of participants**	**RISK:** (low/ high/ unclear)	Low
Rationale of bias rating: Low risk of bias		
**B. Applicability**		
**Concern that the included participants and setting do not match the review question**	**CONCERN:** (low/ high/ unclear)	Low
Rationale of applicability rating: Match the review question		

DOMAIN 2: Predictors		
**A. Risk of Bias**		
2.1 Were predictors defined and assessed in a similar way for all participants?		Yes
2.2 Were predictor assessments made without knowledge of outcome data?		Yes
2.3 Are all predictors available at the time the model is intended to be used?		Yes
**Risk of bias introduced by predictors or their assessment**	**RISK:** (low/ high/ unclear)	Low
Rationale of bias rating: Low risk of bias		
**B. Applicability**		
Concern that the definition, assessment or timing of predictors in the model do not match the review question	**CONCERN:** (low/ high/ unclear)	Low
Rationale of applicability rating: Match the review question		

DOMAIN 3: Outcome		
**A. Risk of Bias**		
3.1 Was the outcome determined appropriately?		Yes
3.2 Was a pre-specified or standard outcome definition used?		Yes
3.3 Were predictors excluded from the outcome definition?		Yes
3.4 Was the outcome defined and determined in a similar way for all participants?		Yes
3.5 Was the outcome determined without knowledge of predictor information?		Yes
3.6 Was the time interval between predictor assessment and outcome determination appropriate?		Yes
**Risk of bias introduced by the outcome or its determination**	**RISK:** (low/ high/ unclear)	Low
Rationale of bias rating: Low risk of bias		
**B. Applicability**		
**Concern that the outcome, its definition, timing or determination do not match the review question**	**CONCERN:** (low/ high/ unclear)	Low
Rationale of applicability rating: Match the review question		

DOMAIN 4: Analysis		
**Risk of Bias**		
4.1 Were there a reasonable number of participants with the outcome?		Yes
4.2 Were continuous and categorical predictors handled appropriately?		Yes
4.3 Were all enrolled participants included in the analysis?		Yes
4.4 Were participants with missing data handled appropriately?		No
4.5 Was selection of predictors based on univariable analysis avoided?		Not applicable
4.6 Were complexities in the data (e.g. censoring, competing risks, sampling of controls) accounted for appropriately?		Yes
4.7 Were relevant model performance measures evaluated appropriately?		No information
4.8 Were model overfitting and optimism in model performance accounted for?		Not applicable
4.9 Do predictors and their assigned weights in the final model correspond to the results from multivariable analysis?		Not applicable
**Risk of bias introduced by the analysis**	**RISK:** (low/ high/ unclear)	High
Rationale of bias rating: Patients with missing data were excluded		

Fan et al.

DOMAIN 1: Participants		
**A. Risk of Bias**		
1.1 Were appropriate data sources used, e.g. cohort, RCT or nested case-control study data?		Yes
1.2 Were all inclusions and exclusions of participants appropriate?		No information
**Risk of bias introduced by selection of participants**	**RISK:** (low/ high/ unclear)	Unclear
Rationale of bias rating: The exclusions of participants were not specified		
**B. Applicability**		
**Concern that the included participants and setting do not match the review question**	**CONCERN:** (low/ high/ unclear)	Low
Rationale of applicability rating: Match the review question		

DOMAIN 2: Predictors		
**A. Risk of Bias**		
2.1 Were predictors defined and assessed in a similar way for all participants?		Yes
2.2 Were predictor assessments made without knowledge of outcome data?		Yes
2.3 Are all predictors available at the time the model is intended to be used?		Yes
**Risk of bias introduced by predictors or their assessment**	**RISK:** (low/ high/ unclear)	Low
Rationale of bias rating: Low risk of bias		
**B. Applicability**		
Concern that the definition, assessment or timing of predictors in the model do not match the review question	**CONCERN:** (low/ high/ unclear)	Low
Rationale of applicability rating: Match the review question		

DOMAIN 3: Outcome		
**A. Risk of Bias**		
3.1 Was the outcome determined appropriately?		Yes
3.2 Was a pre-specified or standard outcome definition used?		Yes
3.3 Were predictors excluded from the outcome definition?		Yes
3.4 Was the outcome defined and determined in a similar way for all participants?		Yes
3.5 Was the outcome determined without knowledge of predictor information?		Yes
3.6 Was the time interval between predictor assessment and outcome determination appropriate?		Yes
**Risk of bias introduced by the outcome or its determination**	**RISK:** (low/ high/ unclear)	Low
Rationale of bias rating: Low risk of bias		
**B. Applicability**		
**Concern that the outcome, its definition, timing or determination do not match the review question**	**CONCERN:** (low/ high/ unclear)	Low
Rationale of applicability rating: Match the review question		

DOMAIN 4: Analysis		
**Risk of Bias**		
4.1 Were there a reasonable number of participants with the outcome?		Yes
4.2 Were continuous and categorical predictors handled appropriately?		Yes
4.3 Were all enrolled participants included in the analysis?		Yes
4.4 Were participants with missing data handled appropriately?		No
4.5 Was selection of predictors based on univariable analysis avoided?		Not applicable
4.6 Were complexities in the data (e.g. censoring, competing risks, sampling of controls) accounted for appropriately?		Yes
4.7 Were relevant model performance measures evaluated appropriately?		No information
4.8 Were model overfitting and optimism in model performance accounted for?		Not applicable
4.9 Do predictors and their assigned weights in the final model correspond to the results from multivariable analysis?		Not applicable
**Risk of bias introduced by the analysis**	**RISK:** (low/ high/ unclear)	High
Rationale of bias rating: Patients with missing data were excluded		

Gidari et al.

DOMAIN 1: Participants		
**A. Risk of Bias**		
1.1 Were appropriate data sources used, e.g. cohort, RCT or nested case-control study data?		Yes
1.2 Were all inclusions and exclusions of participants appropriate?		Yes
**Risk of bias introduced by selection of participants**	**RISK:** (low/ high/ unclear)	Low
Rationale of bias rating: Low risk of bias		
**B. Applicability**		
**Concern that the included participants and setting do not match the review question**	**CONCERN:** (low/ high/ unclear)	Low
Rationale of applicability rating: Match the review question		

DOMAIN 2: Predictors		
**A. Risk of Bias**		
2.1 Were predictors defined and assessed in a similar way for all participants?		Yes
2.2 Were predictor assessments made without knowledge of outcome data?		Yes
2.3 Are all predictors available at the time the model is intended to be used?		Yes
**Risk of bias introduced by predictors or their assessment**	**RISK:** (low/ high/ unclear)	Low
Rationale of bias rating: Low risk of bias		
**B. Applicability**		
Concern that the definition, assessment or timing of predictors in the model do not match the review question	**CONCERN:** (low/ high/ unclear)	Low
Rationale of applicability rating: Match the review question		

DOMAIN 3: Outcome		
**A. Risk of Bias**		
3.1 Was the outcome determined appropriately?		Yes
3.2 Was a pre-specified or standard outcome definition used?		Yes
3.3 Were predictors excluded from the outcome definition?		Yes
3.4 Was the outcome defined and determined in a similar way for all participants?		Yes
3.5 Was the outcome determined without knowledge of predictor information?		Yes
3.6 Was the time interval between predictor assessment and outcome determination appropriate?		Yes
**Risk of bias introduced by the outcome or its determination**	**RISK:** (low/ high/ unclear)	Low
Rationale of bias rating: Low risk of bias		
**B. Applicability**		
**Concern that the outcome, its definition, timing or determination do not match the review question**	**CONCERN:** (low/ high/ unclear)	Low
Rationale of applicability rating: Match the review question		

**DOMAIN 4: Analysis**		
**Risk of Bias**		
4.1 Were there a reasonable number of participants with the outcome?		No
4.2 Were continuous and categorical predictors handled appropriately?		Yes
4.3 Were all enrolled participants included in the analysis?		Yes
4.4 Were participants with missing data handled appropriately?		No information
4.5 Was selection of predictors based on univariable analysis avoided?		Not applicable
4.6 Were complexities in the data (e.g. censoring, competing risks, sampling of controls) accounted for appropriately?		Yes
4.7 Were relevant model performance measures evaluated appropriately?		No information
4.8 Were model overfitting and optimism in model performance accounted for?		Not applicable
4.9 Do predictors and their assigned weights in the final model correspond to the results from multivariable analysis?		Not applicable
**Risk of bias introduced by the analysis**	**RISK:** (low/ high/ unclear)	High
Rationale of bias rating:<br>Sample size was less than 100		

Holten et al.

DOMAIN 1: Participants		
**A. Risk of Bias**		
1.1 Were appropriate data sources used, e.g. cohort, RCT or nested case-control study data?		Yes
1.2 Were all inclusions and exclusions of participants appropriate?		Yes
**Risk of bias introduced by selection of participants**	**RISK:** (low/ high/ unclear)	Low
Rationale of bias rating: Low risk of bias		
**B. Applicability**		
**Concern that the included participants and setting do not match the review question**	**CONCERN:** (low/ high/ unclear)	Low
Rationale of applicability rating: Match the review question		

DOMAIN 2: Predictors		
**A. Risk of Bias**		
2.1 Were predictors defined and assessed in a similar way for all participants?		Yes
2.2 Were predictor assessments made without knowledge of outcome data?		Yes
2.3 Are all predictors available at the time the model is intended to be used?		Yes
**Risk of bias introduced by predictors or their assessment**	**RISK:** (low/ high/ unclear)	Low
Rationale of bias rating: Low risk of bias		
**B. Applicability**		
Concern that the definition, assessment or timing of predictors in the model do not match the review question	**CONCERN:** (low/ high/ unclear)	Low
Rationale of applicability rating: Match the review question		

DOMAIN 3: Outcome		
**A. Risk of Bias**		
3.1 Was the outcome determined appropriately?		Yes
3.2 Was a pre-specified or standard outcome definition used?		Yes
3.3 Were predictors excluded from the outcome definition?		Yes
3.4 Was the outcome defined and determined in a similar way for all participants?		Yes
3.5 Was the outcome determined without knowledge of predictor information?		Yes
3.6 Was the time interval between predictor assessment and outcome determination appropriate?		Yes
**Risk of bias introduced by the outcome or its determination**	**RISK:** (low/ high/ unclear)	Low
Rationale of bias rating: Low risk of bias		
**B. Applicability**		
**Concern that the outcome, its definition, timing or determination do not match the review question**	**CONCERN:** (low/ high/ unclear)	Low
Rationale of applicability rating: Match the review question		

DOMAIN 4: Analysis		
**Risk of Bias**		
4.1 Were there a reasonable number of participants with the outcome?		Yes
4.2 Were continuous and categorical predictors handled appropriately?		Yes
4.3 Were all enrolled participants included in the analysis?		Yes
4.4 Were participants with missing data handled appropriately?		No information
4.5 Was selection of predictors based on univariable analysis avoided?		Not applicable
4.6 Were complexities in the data (e.g. censoring, competing risks, sampling of controls) accounted for appropriately?		Yes
4.7 Were relevant model performance measures evaluated appropriately?		No information
4.8 Were model overfitting and optimism in model performance accounted for?		Not applicable
4.9 Do predictors and their assigned weights in the final model correspond to the results from multivariable analysis?		Not applicable
**Risk of bias introduced by the analysis**	**RISK:** (low/ high/ unclear)	Unclear
Rationale of bias rating: Did not report the missing data and the calibration of NEWS		

Ihle-Hansen et al.

DOMAIN 1: Participants		
**A. Risk of Bias**		
1.1 Were appropriate data sources used, e.g. cohort, RCT or nested case-control study data?		Yes
1.2 Were all inclusions and exclusions of participants appropriate?		Yes
**Risk of bias introduced by selection of participants**	**RISK:** (low/ high/ unclear)	Low
Rationale of bias rating:<br>Low risk of bias		
**B. Applicability**		
**Concern that the included participants and setting do not match the review question**	**CONCERN:** (low/ high/ unclear)	Low
Rationale of applicability rating:<br>Match the review question		

DOMAIN 2: Predictors		
**A. Risk of Bias**		
2.1 Were predictors defined and assessed in a similar way for all participants?		Yes
2.2 Were predictor assessments made without knowledge of outcome data?		Yes
2.3 Are all predictors available at the time the model is intended to be used?		Yes
**Risk of bias introduced by predictors or their assessment**	**RISK:** (low/ high/ unclear)	Low
Rationale of bias rating:<br>Low risk of bias		
**B. Applicability**		
Concern that the definition, assessment or timing of predictors in the model do not match the review question	**CONCERN:** (low/ high/ unclear)	Low
Rationale of applicability rating:<br>Match the review question		

DOMAIN 3: Outcome		
**A. Risk of Bias**		
3.1 Was the outcome determined appropriately?		Yes
3.2 Was a pre-specified or standard outcome definition used?		Yes
3.3 Were predictors excluded from the outcome definition?		Yes
3.4 Was the outcome defined and determined in a similar way for all participants?		Yes
3.5 Was the outcome determined without knowledge of predictor information?		Yes
3.6 Was the time interval between predictor assessment and outcome determination appropriate?		No information
**Risk of bias introduced by the outcome or its determination**	**RISK:** (low/ high/ unclear)	Unclear
Rationale of bias rating:<br>The time interval between predictor assessment and outcome determination was not specified		
**B. Applicability**		
**Concern that the outcome, its definition, timing or determination do not match the review question**	**CONCERN:** (low/ high/ unclear)	Low
Rationale of applicability rating:<br>Match the review question		

DOMAIN 4: Analysis		
**Risk of Bias**		
4.1 Were there a reasonable number of participants with the outcome?		No
4.2 Were continuous and categorical predictors handled appropriately?		Yes
4.3 Were all enrolled participants included in the analysis?		Yes
4.4 Were participants with missing data handled appropriately?		No information
4.5 Was selection of predictors based on univariable analysis avoided?		Not applicable
4.6 Were complexities in the data (e.g. censoring, competing risks, sampling of controls) accounted for appropriately?		Yes
4.7 Were relevant model performance measures evaluated appropriately?		No information
4.8 Were model overfitting and optimism in model performance accounted for?		Not applicable
4.9 Do predictors and their assigned weights in the final model correspond to the results from multivariable analysis?		Not applicable
**Risk of bias introduced by the analysis**	**RISK:** (low/ high/ unclear)	High
Rationale of bias rating: Sample size was less than 100		

Jang et al.

DOMAIN 1: Participants		
**A. Risk of Bias**		
1.1 Were appropriate data sources used, e.g. cohort, RCT or nested case-control study data?		Yes
1.2 Were all inclusions and exclusions of participants appropriate?		Yes
**Risk of bias introduced by selection of participants**	**RISK:** (low/ high/ unclear)	Low
Rationale of bias rating:<br>Low risk of bias		
**B. Applicability**		
**Concern that the included participants and setting do not match the review question**	**CONCERN:** (low/ high/ unclear)	Low
Rationale of applicability rating:<br>Match the review question		

DOMAIN 2: Predictors		
**A. Risk of Bias**		
2.1 Were predictors defined and assessed in a similar way for all participants?		Yes
2.2 Were predictor assessments made without knowledge of outcome data?		Yes
2.3 Are all predictors available at the time the model is intended to be used?		Yes
**Risk of bias introduced by predictors or their assessment**	**RISK:** (low/ high/ unclear)	Low
Rationale of bias rating:<br>Low risk of bias		
**B. Applicability**		
Concern that the definition, assessment or timing of predictors in the model do not match the review question	**CONCERN:** (low/ high/ unclear)	Low
Rationale of applicability rating:<br>Match the review question		

DOMAIN 3: Outcome		
**A. Risk of Bias**		
3.1 Was the outcome determined appropriately?		Yes
3.2 Was a pre-specified or standard outcome definition used?		Yes
3.3 Were predictors excluded from the outcome definition?		Yes
3.4 Was the outcome defined and determined in a similar way for all participants?		Yes
3.5 Was the outcome determined without knowledge of predictor information?		Yes
3.6 Was the time interval between predictor assessment and outcome determination appropriate?		No information
**Risk of bias introduced by the outcome or its determination**	**RISK:** (low/ high/ unclear)	Unclear
Rationale of bias rating:<br>The time interval between predictor assessment and outcome determination was not specified		
**B. Applicability**		
**Concern that the outcome, its definition, timing or determination do not match the review question**	**CONCERN:** (low/ high/ unclear)	Unclear
Rationale of applicability rating:<br>The time interval between predictor assessment and outcome determination was not specified		

**DOMAIN 4: Analysis**		
**Risk of Bias**		
4.1 Were there a reasonable number of participants with the outcome?		Yes
4.2 Were continuous and categorical predictors handled appropriately?		Yes
4.3 Were all enrolled participants included in the analysis?		Yes
4.4 Were participants with missing data handled appropriately?		No information
4.5 Was selection of predictors based on univariable analysis avoided?		Not applicable
4.6 Were complexities in the data (e.g. censoring, competing risks, sampling of controls) accounted for appropriately?		Yes
4.7 Were relevant model performance measures evaluated appropriately?		No information
4.8 Were model overfitting and optimism in model performance accounted for?		Not applicable
4.9 Do predictors and their assigned weights in the final model correspond to the results from multivariable analysis?		Not applicable
**Risk of bias introduced by the analysis**	**RISK:** (low/ high/ unclear)	High
Rationale of bias rating: Did not report the missing data and the calibration of NEWS		

Liu et al.

DOMAIN 1: Participants		
**A. Risk of Bias**		
1.1 Were appropriate data sources used, e.g. cohort, RCT or nested case-control study data?		Yes
1.2 Were all inclusions and exclusions of participants appropriate?		Yes
**Risk of bias introduced by selection of participants**	**RISK:** (low/ high/ unclear)	Low
Rationale of bias rating: Low risk of bias		
**B. Applicability**		
**Concern that the included participants and setting do not match the review question**	**CONCERN:** (low/ high/ unclear)	Low
Rationale of applicability rating: Match the review question		

DOMAIN 2: Predictors		
**A. Risk of Bias**		
2.1 Were predictors defined and assessed in a similar way for all participants?		Yes
2.2 Were predictor assessments made without knowledge of outcome data?		Yes
2.3 Are all predictors available at the time the model is intended to be used?		Yes
**Risk of bias introduced by predictors or their assessment**	**RISK:** (low/ high/ unclear)	Low
Rationale of bias rating: Low risk of bias		
**B. Applicability**		
Concern that the definition, assessment or timing of predictors in the model do not match the review question	**CONCERN:** (low/ high/ unclear)	Low
Rationale of applicability rating: Match the review question		

DOMAIN 3: Outcome		
**A. Risk of Bias**		
3.1 Was the outcome determined appropriately?		Yes
3.2 Was a pre-specified or standard outcome definition used?		Yes
3.3 Were predictors excluded from the outcome definition?		Yes
3.4 Was the outcome defined and determined in a similar way for all participants?		Yes
3.5 Was the outcome determined without knowledge of predictor information?		Yes
3.6 Was the time interval between predictor assessment and outcome determination appropriate?		Yes
**Risk of bias introduced by the outcome or its determination**	**RISK:** (low/ high/ unclear)	Low
Rationale of bias rating: Low risk of bias		
**B. Applicability**		
**Concern that the outcome, its definition, timing or determination do not match the review question**	**CONCERN:** (low/ high/ unclear)	Low
Rationale of applicability rating: Match the review question		

DOMAIN 4: Analysis		
**Risk of Bias**		
4.1 Were there a reasonable number of participants with the outcome?		Yes
4.2 Were continuous and categorical predictors handled appropriately?		Yes
4.3 Were all enrolled participants included in the analysis?		Yes
4.4 Were participants with missing data handled appropriately?		Yes
4.5 Was selection of predictors based on univariable analysis avoided?		Not applicable
4.6 Were complexities in the data (e.g. censoring, competing risks, sampling of controls) accounted for appropriately?		Yes
4.7 Were relevant model performance measures evaluated appropriately?		Yes
4.8 Were model overfitting and optimism in model performance accounted for?		Not applicable
4.9 Do predictors and their assigned weights in the final model correspond to the results from multivariable analysis?		Not applicable
**Risk of bias introduced by the analysis**	**RISK:** (low/ high/ unclear)	Low
Rationale of bias rating: Low risk of bias		

Maguire et al.

DOMAIN 1: Participants		
**A. Risk of Bias**		
1.1 Were appropriate data sources used, e.g. cohort, RCT or nested case-control study data?		Yes
1.2 Were all inclusions and exclusions of participants appropriate?		Yes
**Risk of bias introduced by selection of participants**	**RISK:** (low/ high/ unclear)	Low
Rationale of bias rating: Low risk of bias		
**B. Applicability**		
**Concern that the included participants and setting do not match the review question**	**CONCERN:** (low/ high/ unclear)	Low
Rationale of applicability rating: Match the review question		

DOMAIN 2: Predictors		
**A. Risk of Bias**		
2.1 Were predictors defined and assessed in a similar way for all participants?		Yes
2.2 Were predictor assessments made without knowledge of outcome data?		Yes
2.3 Are all predictors available at the time the model is intended to be used?		Yes
**Risk of bias introduced by predictors or their assessment**	**RISK:** (low/ high/ unclear)	Low
Rationale of bias rating: Low risk of bias		
**B. Applicability**		
Concern that the definition, assessment or timing of predictors in the model do not match the review question	**CONCERN:** (low/ high/ unclear)	Low
Rationale of applicability rating: Match the review question		

DOMAIN 3: Outcome		
**A. Risk of Bias**		
3.1 Was the outcome determined appropriately?		Yes
3.2 Was a pre-specified or standard outcome definition used?		Yes
3.3 Were predictors excluded from the outcome definition?		Yes
3.4 Was the outcome defined and determined in a similar way for all participants?		Yes
3.5 Was the outcome determined without knowledge of predictor information?		Yes
3.6 Was the time interval between predictor assessment and outcome determination appropriate?		Yes
**Risk of bias introduced by the outcome or its determination**	**RISK:** (low/ high/ unclear)	Low
Rationale of bias rating: Low risk of bias		
**B. Applicability**		
**Concern that the outcome, its definition, timing or determination do not match the review question**	**CONCERN:** (low/ high/ unclear)	Low
Rationale of applicability rating: Match the review question		

DOMAIN 4: Analysis		
**Risk of Bias**		
4.1 Were there a reasonable number of participants with the outcome?		Yes
4.2 Were continuous and categorical predictors handled appropriately?		Yes
4.3 Were all enrolled participants included in the analysis?		Yes
4.4 Were participants with missing data handled appropriately?		No
4.5 Was selection of predictors based on univariable analysis avoided?		Not applicable
4.6 Were complexities in the data (e.g. censoring, competing risks, sampling of controls) accounted for appropriately?		Yes
4.7 Were relevant model performance measures evaluated appropriately?		No information
4.8 Were model overfitting and optimism in model performance accounted for?		Not applicable
4.9 Do predictors and their assigned weights in the final model correspond to the results from multivariable analysis?		Not applicable
**Risk of bias introduced by the analysis**	**RISK:** (low/ high/ unclear)	High
Rationale of bias rating: Patients with missing data were excluded		

Martín-Rodríguez et al.

DOMAIN 1: Participants		
**A. Risk of Bias**		
1.1 Were appropriate data sources used, e.g. cohort, RCT or nested case-control study data?		Yes
1.2 Were all inclusions and exclusions of participants appropriate?		Yes
**Risk of bias introduced by selection of participants**	**RISK:** (low/ high/ unclear)	Low
Rationale of bias rating: Low risk of bias		
**B. Applicability**		
**Concern that the included participants and setting do not match the review question**	**CONCERN:** (low/ high/ unclear)	Low
Rationale of applicability rating: Match the review question		

DOMAIN 2: Predictors		
**A. Risk of Bias**		
2.1 Were predictors defined and assessed in a similar way for all participants?		Yes
2.2 Were predictor assessments made without knowledge of outcome data?		Yes
2.3 Are all predictors available at the time the model is intended to be used?		Yes
**Risk of bias introduced by predictors or their assessment**	**RISK:** (low/ high/ unclear)	Low
Rationale of bias rating: Low risk of bias		
**B. Applicability**		
Concern that the definition, assessment or timing of predictors in the model do not match the review question	**CONCERN:** (low/ high/ unclear)	Low
Rationale of applicability rating: Match the review question		

DOMAIN 3: Outcome		
**A. Risk of Bias**		
3.1 Was the outcome determined appropriately?		Yes
3.2 Was a pre-specified or standard outcome definition used?		Yes
3.3 Were predictors excluded from the outcome definition?		Yes
3.4 Was the outcome defined and determined in a similar way for all participants?		Yes
3.5 Was the outcome determined without knowledge of predictor information?		Yes
3.6 Was the time interval between predictor assessment and outcome determination appropriate?		Yes
**Risk of bias introduced by the outcome or its determination**	**RISK:** (low/ high/ unclear)	Low
Rationale of bias rating: Low risk of bias		
**B. Applicability**		
**Concern that the outcome, its definition, timing or determination do not match the review question**	**CONCERN:** (low/ high/ unclear)	Low
Rationale of applicability rating: Match the review question		

DOMAIN 4: Analysis		
**Risk of Bias**		
4.1 Were there a reasonable number of participants with the outcome?		Yes
4.2 Were continuous and categorical predictors handled appropriately?		Yes
4.3 Were all enrolled participants included in the analysis?		Yes
4.4 Were participants with missing data handled appropriately?		Yes
4.5 Was selection of predictors based on univariable analysis avoided?		Not applicable
4.6 Were complexities in the data (e.g. censoring, competing risks, sampling of controls) accounted for appropriately?		Yes
4.7 Were relevant model performance measures evaluated appropriately?		Yes
4.8 Were model overfitting and optimism in model performance accounted for?		Not applicable
4.9 Do predictors and their assigned weights in the final model correspond to the results from multivariable analysis?		Not applicable
**Risk of bias introduced by the analysis**	**RISK:** (low/ high/ unclear)	Low
Rationale of bias rating: Missing values were replaced using the mode of the variable		

Myrstad et al.

DOMAIN 1: Participants		
**A. Risk of Bias**		
1.1 Were appropriate data sources used, e.g. cohort, RCT or nested case-control study data?		Yes
1.2 Were all inclusions and exclusions of participants appropriate?		Yes
**Risk of bias introduced by selection of participants**	**RISK:** (low/ high/ unclear)	Low
Rationale of bias rating: Low risk of bias		
**B. Applicability**		
**Concern that the included participants and setting do not match the review question**	**CONCERN:** (low/ high/ unclear)	Low
Rationale of applicability rating: Match the review question		

DOMAIN 2: Predictors		
**A. Risk of Bias**		
2.1 Were predictors defined and assessed in a similar way for all participants?		Yes
2.2 Were predictor assessments made without knowledge of outcome data?		Yes
2.3 Are all predictors available at the time the model is intended to be used?		Yes
**Risk of bias introduced by predictors or their assessment**	**RISK:** (low/ high/ unclear)	Low
Rationale of bias rating: Low risk of bias		
**B. Applicability**		
Concern that the definition, assessment or timing of predictors in the model do not match the review question	**CONCERN:** (low/ high/ unclear)	Low
Rationale of applicability rating: Match the review question		

DOMAIN 3: Outcome		
**A. Risk of Bias**		
3.1 Was the outcome determined appropriately?		Yes
3.2 Was a pre-specified or standard outcome definition used?		Yes
3.3 Were predictors excluded from the outcome definition?		Yes
3.4 Was the outcome defined and determined in a similar way for all participants?		Yes
3.5 Was the outcome determined without knowledge of predictor information?		Yes
3.6 Was the time interval between predictor assessment and outcome determination appropriate?		Yes
**Risk of bias introduced by the outcome or its determination**	**RISK:** (low/ high/ unclear)	Low
Rationale of bias rating: Low risk of bias		
**B. Applicability**		
**Concern that the outcome, its definition, timing or determination do not match the review question**	**CONCERN:** (low/ high/ unclear)	Low
Rationale of applicability rating: Match the review question		

DOMAIN 4: Analysis		
**Risk of Bias**		
4.1 Were there a reasonable number of participants with the outcome?		No
4.2 Were continuous and categorical predictors handled appropriately?		Yes
4.3 Were all enrolled participants included in the analysis?		Yes
4.4 Were participants with missing data handled appropriately?		Yes
4.5 Was selection of predictors based on univariable analysis avoided?		Not applicable
4.6 Were complexities in the data (e.g. censoring, competing risks, sampling of controls) accounted for appropriately?		Yes
4.7 Were relevant model performance measures evaluated appropriately?		No information
4.8 Were model overfitting and optimism in model performance accounted for?		Not applicable
4.9 Do predictors and their assigned weights in the final model correspond to the results from multivariable analysis?		Not applicable
**Risk of bias introduced by the analysis**	**RISK:** (low/ high/ unclear)	High
Rationale of bias rating: Sample size was less than 100		

Pokeerbux et al.

DOMAIN 1: Participants		
**A. Risk of Bias**		
1.1 Were appropriate data sources used, e.g. cohort, RCT or nested case-control study data?		Yes
1.2 Were all inclusions and exclusions of participants appropriate?		Yes
**Risk of bias introduced by selection of participants**	**RISK:** (low/ high/ unclear)	Low
Rationale of bias rating: Low risk of bias		
**B. Applicability**		
**Concern that the included participants and setting do not match the review question**	**CONCERN:** (low/ high/ unclear)	Low
Rationale of applicability rating: Match the review question		

DOMAIN 2: Predictors		
**A. Risk of Bias**		
2.1 Were predictors defined and assessed in a similar way for all participants?		Yes
2.2 Were predictor assessments made without knowledge of outcome data?		Yes
2.3 Are all predictors available at the time the model is intended to be used?		Yes
**Risk of bias introduced by predictors or their assessment**	**RISK:** (low/ high/ unclear)	Low
Rationale of bias rating: Low risk of bias		
**B. Applicability**		
Concern that the definition, assessment or timing of predictors in the model do not match the review question	**CONCERN:** (low/ high/ unclear)	Low
Rationale of applicability rating: Match the review question		

DOMAIN 3: Outcome		
**A. Risk of Bias**		
3.1 Was the outcome determined appropriately?		Yes
3.2 Was a pre-specified or standard outcome definition used?		Yes
3.3 Were predictors excluded from the outcome definition?		Yes
3.4 Was the outcome defined and determined in a similar way for all participants?		Yes
3.5 Was the outcome determined without knowledge of predictor information?		Yes
3.6 Was the time interval between predictor assessment and outcome determination appropriate?		Yes
**Risk of bias introduced by the outcome or its determination**	**RISK:** (low/ high/ unclear)	Low
Rationale of bias rating: Low risk of bias		
**B. Applicability**		
**Concern that the outcome, its definition, timing or determination do not match the review question**	**CONCERN:** (low/ high/ unclear)	Low
Rationale of applicability rating: Match the review question		

DOMAIN 4: Analysis		
**Risk of Bias**		
4.1 Were there a reasonable number of participants with the outcome?		Yes
4.2 Were continuous and categorical predictors handled appropriately?		Yes
4.3 Were all enrolled participants included in the analysis?		Yes
4.4 Were participants with missing data handled appropriately?		No information
4.5 Was selection of predictors based on univariable analysis avoided?		Not applicable
4.6 Were complexities in the data (e.g. censoring, competing risks, sampling of controls) accounted for appropriately?		Yes
4.7 Were relevant model performance measures evaluated appropriately?		No information
4.8 Were model overfitting and optimism in model performance accounted for?		Not applicable
4.9 Do predictors and their assigned weights in the final model correspond to the results from multivariable analysis?		Not applicable
**Risk of bias introduced by the analysis**	**RISK:** (low/ high/ unclear)	Unclear
Rationale of bias rating: Did not report the missing data and the calibration of NEWS		

Prower et al.

DOMAIN 1: Participants		
**A. Risk of Bias**		
1.1 Were appropriate data sources used, e.g. cohort, RCT or nested case-control study data?		Yes
1.2 Were all inclusions and exclusions of participants appropriate?		Yes
**Risk of bias introduced by selection of participants**	**RISK:** (low/ high/ unclear)	Low
Rationale of bias rating: Low risk of bias		
**B. Applicability**		
**Concern that the included participants and setting do not match the review question**	**CONCERN:** (low/ high/ unclear)	Low
Rationale of applicability rating: Match the review question		

DOMAIN 2: Predictors		
**A. Risk of Bias**		
2.1 Were predictors defined and assessed in a similar way for all participants?		Yes
2.2 Were predictor assessments made without knowledge of outcome data?		Yes
2.3 Are all predictors available at the time the model is intended to be used?		Yes
**Risk of bias introduced by predictors or their assessment**	**RISK:** (low/ high/ unclear)	Low
Rationale of bias rating: Low risk of bias		
**B. Applicability**		
Concern that the definition, assessment or timing of predictors in the model do not match the review question	**CONCERN:** (low/ high/ unclear)	Low
Rationale of applicability rating: Match the review question		

DOMAIN 3: Outcome		
**A. Risk of Bias**		
3.1 Was the outcome determined appropriately?		Yes
3.2 Was a pre-specified or standard outcome definition used?		Yes
3.3 Were predictors excluded from the outcome definition?		Yes
3.4 Was the outcome defined and determined in a similar way for all participants?		Yes
3.5 Was the outcome determined without knowledge of predictor information?		Yes
3.6 Was the time interval between predictor assessment and outcome determination appropriate?		Yes
**Risk of bias introduced by the outcome or its determination**	**RISK:** (low/ high/ unclear)	Low
Rationale of bias rating: Low risk of bias		
**B. Applicability**		
**Concern that the outcome, its definition, timing or determination do not match the review question**	**CONCERN:** (low/ high/ unclear)	Low
Rationale of applicability rating: Match the review question		

DOMAIN 4: Analysis		
**Risk of Bias**		
4.1 Were there a reasonable number of participants with the outcome?		Yes
4.2 Were continuous and categorical predictors handled appropriately?		Yes
4.3 Were all enrolled participants included in the analysis?		Yes
4.4 Were participants with missing data handled appropriately?		No information
4.5 Was selection of predictors based on univariable analysis avoided?		Not applicable
4.6 Were complexities in the data (e.g. censoring, competing risks, sampling of controls) accounted for appropriately?		Yes
4.7 Were relevant model performance measures evaluated appropriately?		No information
4.8 Were model overfitting and optimism in model performance accounted for?		Not applicable
4.9 Do predictors and their assigned weights in the final model correspond to the results from multivariable analysis?		Not applicable
**Risk of bias introduced by the analysis**	**RISK:** (low/ high/ unclear)	Unclear
Rationale of bias rating: Did not report the missing data and the calibration of NEWS		

Richardson et al.

DOMAIN 1: Participants		
**A. Risk of Bias**		
1.1 Were appropriate data sources used, e.g. cohort, RCT or nested case-control study data?		Yes
1.2 Were all inclusions and exclusions of participants appropriate?		Yes
**Risk of bias introduced by selection of participants**	**RISK:** (low/ high/ unclear)	Low
Rationale of bias rating:<br>Low risk of bias		
**B. Applicability**		
**Concern that the included participants and setting do not match the review question**	**CONCERN:** (low/ high/ unclear)	Low
Rationale of applicability rating:<br>Match the review question		

DOMAIN 2: Predictors		
**A. Risk of Bias**		
2.1 Were predictors defined and assessed in a similar way for all participants?		Yes
2.2 Were predictor assessments made without knowledge of outcome data?		Yes
2.3 Are all predictors available at the time the model is intended to be used?		Yes
**Risk of bias introduced by predictors or their assessment**	**RISK:** (low/ high/ unclear)	Low
Rationale of bias rating:<br>Low risk of bias		
**B. Applicability**		
Concern that the definition, assessment or timing of predictors in the model do not match the review question	**CONCERN:** (low/ high/ unclear)	Low
Rationale of applicability rating:<br>Match the review question		

DOMAIN 3: Outcome		
**A. Risk of Bias**		
3.1 Was the outcome determined appropriately?		Yes
3.2 Was a pre-specified or standard outcome definition used?		Yes
3.3 Were predictors excluded from the outcome definition?		Yes
3.4 Was the outcome defined and determined in a similar way for all participants?		Yes
3.5 Was the outcome determined without knowledge of predictor information?		Yes
3.6 Was the time interval between predictor assessment and outcome determination appropriate?		Yes
**Risk of bias introduced by the outcome or its determination**	**RISK:** (low/ high/ unclear)	Low
Rationale of bias rating:<br>Low risk of bias		
**B. Applicability**		
**Concern that the outcome, its definition, timing or determination do not match the review question**	**CONCERN:** (low/ high/ unclear)	Low
Rationale of applicability rating:<br>Match the review question		

DOMAIN 4: Analysis		
**Risk of Bias**		
4.1 Were there a reasonable number of participants with the outcome?		Yes
4.2 Were continuous and categorical predictors handled appropriately?		Yes
4.3 Were all enrolled participants included in the analysis?		Yes
4.4 Were participants with missing data handled appropriately?		No
4.5 Was selection of predictors based on univariable analysis avoided?		Not applicable
4.6 Were complexities in the data (e.g. censoring, competing risks, sampling of controls) accounted for appropriately?		Yes
4.7 Were relevant model performance measures evaluated appropriately?		No information
4.8 Were model overfitting and optimism in model performance accounted for?		Not applicable
4.9 Do predictors and their assigned weights in the final model correspond to the results from multivariable analysis?		Not applicable
**Risk of bias introduced by the analysis**	**RISK:** (low/ high/ unclear)	High
Rationale of bias rating:<br>Patients with missing data were excluded		

Su et al.

DOMAIN 1: Participants		
**A. Risk of Bias**		
1.1 Were appropriate data sources used, e.g. cohort, RCT or nested case-control study data?		Yes
1.2 Were all inclusions and exclusions of participants appropriate?		Yes
**Risk of bias introduced by selection of participants**	**RISK:** (low/ high/ unclear)	Low
Rationale of bias rating: Low risk of bias		
**B. Applicability**		
**Concern that the included participants and setting do not match the review question**	**CONCERN:** (low/ high/ unclear)	Low
Rationale of applicability rating: Match the review question		

DOMAIN 2: Predictors		
**A. Risk of Bias**		
2.1 Were predictors defined and assessed in a similar way for all participants?		Yes
2.2 Were predictor assessments made without knowledge of outcome data?		Yes
2.3 Are all predictors available at the time the model is intended to be used?		Yes
**Risk of bias introduced by predictors or their assessment**	**RISK:** (low/ high/ unclear)	Low
Rationale of bias rating: Low risk of bias		
**B. Applicability**		
Concern that the definition, assessment or timing of predictors in the model do not match the review question	**CONCERN:** (low/ high/ unclear)	High
Rationale of applicability rating: The threshold value of NEWS was not consistent with other studies		

DOMAIN 3: Outcome		
**A. Risk of Bias**		
3.1 Was the outcome determined appropriately?		Yes
3.2 Was a pre-specified or standard outcome definition used?		Yes
3.3 Were predictors excluded from the outcome definition?		Yes
3.4 Was the outcome defined and determined in a similar way for all participants?		Yes
3.5 Was the outcome determined without knowledge of predictor information?		Yes
3.6 Was the time interval between predictor assessment and outcome determination appropriate?		Yes
**Risk of bias introduced by the outcome or its determination**	**RISK:** (low/ high/ unclear)	Low
Rationale of bias rating: Low risk of bias		
**B. Applicability**		
**Concern that the outcome, its definition, timing or determination do not match the review question**	**CONCERN:** (low/ high/ unclear)	Low
Rationale of applicability rating: Match the review question		

DOMAIN 4: Analysis		
**Risk of Bias**		
4.1 Were there a reasonable number of participants with the outcome?		Yes
4.2 Were continuous and categorical predictors handled appropriately?		Yes
4.3 Were all enrolled participants included in the analysis?		Yes
4.4 Were participants with missing data handled appropriately?		No information
4.5 Was selection of predictors based on univariable analysis avoided?		Not applicable
4.6 Were complexities in the data (e.g. censoring, competing risks, sampling of controls) accounted for appropriately?		Yes
4.7 Were relevant model performance measures evaluated appropriately?		No information
4.8 Were model overfitting and optimism in model performance accounted for?		Not applicable
4.9 Do predictors and their assigned weights in the final model correspond to the results from multivariable analysis?		Not applicable
**Risk of bias introduced by the analysis**	**RISK:** (low/ high/ unclear)	Unclear
Rationale of bias rating: Did not report the missing data and the calibration of NEWS		
