# Supplementary material for: The Prognostic Accuracy of National Early Warning Score 2 on Predicting Clinical Deterioration for Patients With COVID-19: A Systematic Review and Meta-Analysis
Source: Front Med (Lausanne). 2021 Jul 9;8:699880. doi: 10.3389/fmed.2021.699880 (PMC8298908; doi:10.3389/fmed.2021.699880)

**Supplementary File 6: Publication bias evaluated by Deek’s test**

|  | Coefficient | Standard Error | t | P > \|t\| | 95% CI |
| --- | --- | --- | --- | --- | --- |
| Bias | 15.07436 | 7.461572 | 2.02 | 0.066 | -1.183008, 31.33173 |
| Intercept | 1.104056 | 0.464739 | 2.38 | 0.035 | 0.0914764, 2.116635 |


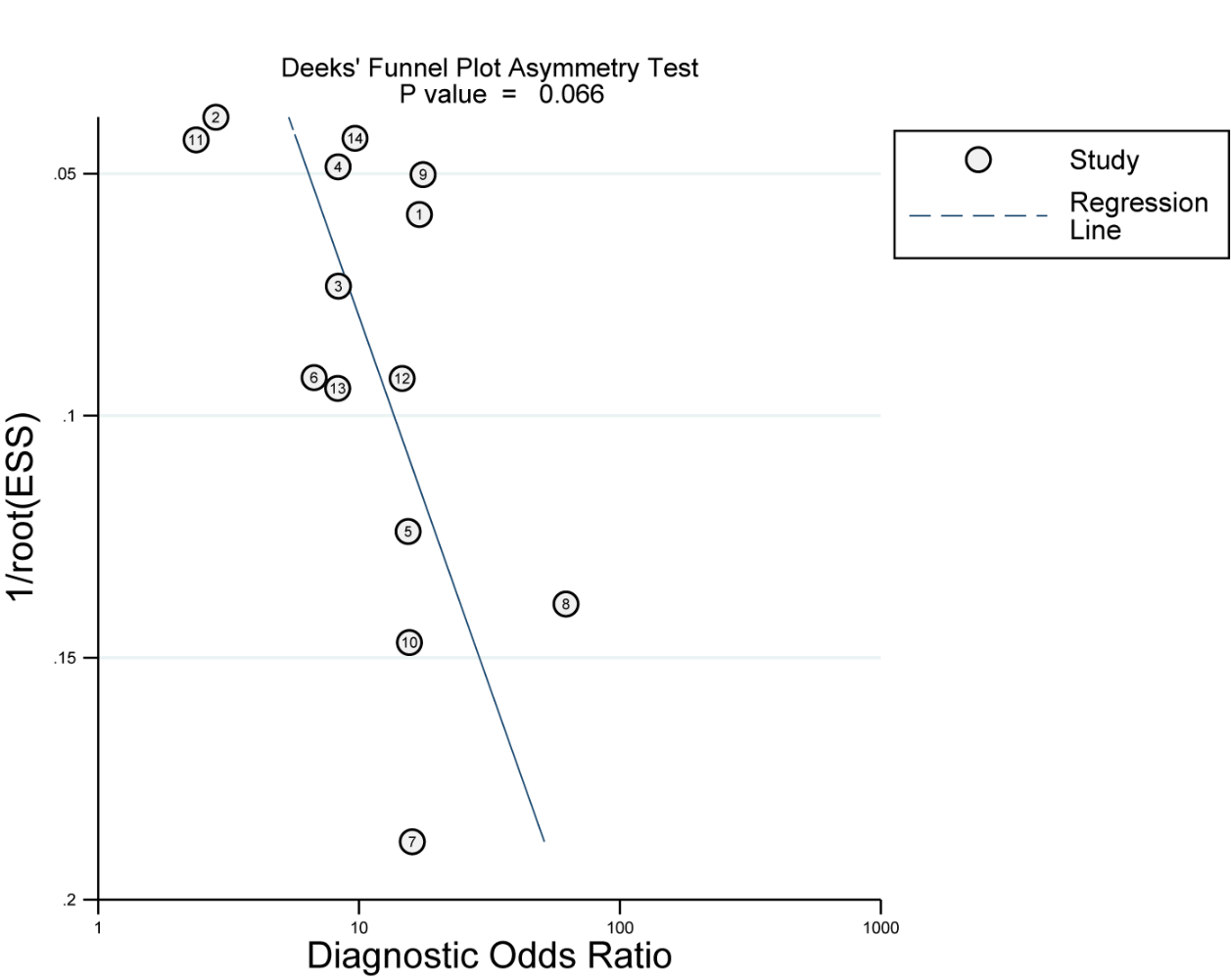

Supplement: Supplementary file 6 [file Table_6.DOCX]
